# Characterization of Fish Serotonin, Dopamine and Norepinephrine Transporters as a Potential Target for Environmental Pharmaceuticals

**DOI:** 10.1021/acs.est.6c04982

**Published:** 2026-08-24

**Authors:** Kikuko Honda, Minguang Han, Fuyuka Mori, Ayaka Morinaga, Yuka Nishimura, Renji Kaneko, Kie Oizumi, Hana Kajiyama, Mariko O. Ihara, Han Zhang, Daisuke Kato, Kenji Toyota, Anke Lange, Charles R. Tyler, Taisen Iguchi, Yuji Mushirobira, Masaki Nagae, Kiyoshi Soyano, Masaru Ihara, Shinichi Miyagawa

**Affiliations:** † Department of Biological Science and Technology, Faculty of Advanced Engineering, Tokyo University of Science, Tokyo 125-8585, Japan; ‡ Department of Environmental Engineering, Graduate School of Engineering, 12918 Kyoto University, Kyoto 615-8246, Japan; § Research Center for Environmental Quality Management, Kyoto University, Shiga 520-0811, Japan; ∥ Faculty of Agriculture and Marine Science, Kochi University, Kochi 783-8502, Japan; ⊥ Key Laboratory of Industrial Ecology and Environmental Engineering (Ministry of Education), Dalian Key Laboratory on Chemicals Risk Control and Pollution Prevention Technology, School of Environmental Science and Technology, 12399 Dalian University of Technology, Dalian, Liaoning 116024, China; # Graduate School of Integrated Sciences for Life, Hiroshima University, Hiroshima 739-8528, Japan; ∇ Biosciences, Faculty of Health and Life Sciences, 3286 University of Exeter, Exeter EX4 4QD, U.K.; ○ Graduate School of Nanobioscience, Yokohama City University, Kanagawa 236-0027, Japan; ◆ Graduate School of Fisheries and Environmental Sciences, Nagasaki University, Nagasaki 852-8521, Japan; ¶ Institute for East China Sea Research, Organization for Marine Science and Technology, Nagasaki University, Nagasaki 851-2213, Japan; †† Division of Biological Fluctuation, Research Institute for Science and Technology (RIST), Tokyo University of Science, Tokyo 125-8585, Japan

**Keywords:** monoamine transporters, environmental pharmaceuticals, antidepressants, medaka, ayu

## Abstract

Antidepressants are environmental contaminants widely prevalent in the environment that can affect aquatic organisms, but their underlying molecular mechanisms and species-specific sensitivities remain poorly understood. To address this knowledge gap, we performed a comprehensive molecular and pharmacological characterization of monoamine transporters (MATs), the primary targets of these pharmaceuticals, from two phylogenetically distinct teleost fish, medaka (*Oryzias latipes*) and ayu (*Plecoglossus altivelis*). We first cloned the cDNAs for two serotonin transporters (SERTa and SERTb), a dopamine transporter (DAT), and a norepinephrine transporter (NET) from both species. Pharmacological assays showed that fish SERTa is substantially more sensitive than SERTb to several antidepressants. While the overall inhibitory profiles of the antidepressants were generally conserved across species, fish SERTa orthologs were consistently and markedly more sensitive to human-targeted pharmaceuticals than the human SERT ortholog. Notably, inhibitory effects were also observed for several pharmaceuticals not traditionally recognized as targeting MATs in human. These molecular mechanisms findings help illustrate the vulnerability of aquatic wildlife to environmentally relevant concentrations of these pharmaceuticals.

## Graphical abstract

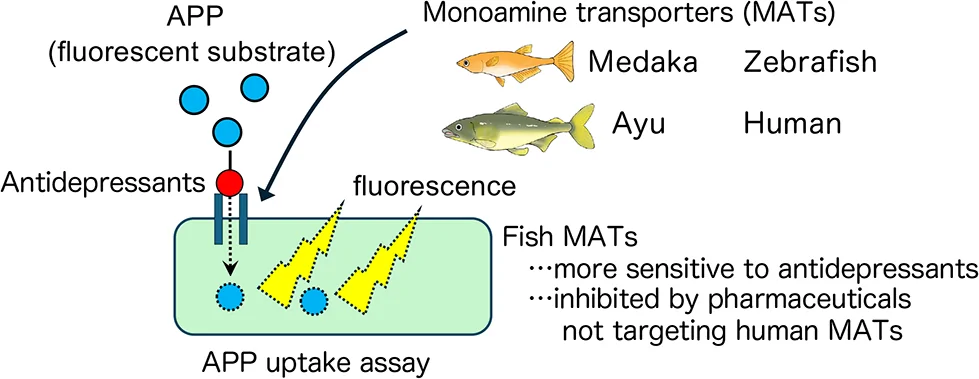

## Introduction

Monoamines, including serotonin (5-hydroxytryptamine; 5-HT), dopamine and norepinephrine, function as key neurotransmitters in a broad range of animals.^1^ Following synaptic release, they are rapidly cleared from the synaptic cleft primarily through reuptake via specific neurotransmitter transporters: the serotonin transporter (SERT: encoded by the solute carrier family 6, member 4 gene, *SLC6A4* in human), dopamine transporter (DAT; *SLC6A3*) and norepinephrine transporter (NET; *SLC6A2*), which belong to the Na/Cl-dependent neurotransmitter transporter gene family.^2^ These monoamine transporters (MATs) can act as a primary target for various antidepressants and antipsychotics, including selective serotonin reuptake inhibitors (SSRI), serotonin-norepinephrine reuptake inhibitors (SNRI), tricyclic antidepressants (TCA) and tetracyclic antidepressants (TeCA). These pharmaceuticals function by inhibiting monoamine reuptake into presynaptic cells thereby increasing neurotransmitter concentrations within the synaptic cleft and modulating neuronal activity.^3,4^

Following human consumption and excretion, a significant portion of pharmaceuticals and their metabolites can enter sewage systems. Due to their diverse molecular properties, not all of them can be effectively removed during wastewater treatment processes, resulting in their discharge into aquatic environment. Consequently, antidepressants such as fluoxetine, sertraline, fluvoxamine, citalopram, venlafaxine, clomipramine, duloxetine, and bupropion, have been detected in environmental waters widely across the globe at concentrations ranging from ng/L to μg/L,^5–11^ raising concerns regarding their potential ecotoxicological impacts on aquatic organisms.^12^ Indeed, many antidepressants have been documented to induce a wide spectrum of toxicological impacts in fish, including neurodevelopmental toxicity, metabolic and endocrine disruption, and physiological impairments.^13,14^ For example, fluoxetine (marketed as Prozac) elicits anxiolytic-like effects in multiple fish species including zebrafish, fathead minnow and medaka, and this is considered as functionally equivalent to the anxiety-reducing effects observed in human patients treated with fluoxetine.^15–18^ While other antidepressants have also been associated with behavioral changes and physiological alternations,^19,20^ it remains unclear whether these effects in fish are mediated through the same molecular pathways as in humans.

Pharmaceuticals are specifically designed to interact with human protein targets and it remains largely unknown whether fish possess orthologous proteins and, if so, to what extent these fish proteins are modulated by human-targeted drugs. Although genes encoding major neurotransmitter pathway components and pharmaceutical targets are generally conserved across vertebrates,^21–24^ the gene repertoire and functional roles of MATs in most fish species are still poorly understood. To rigorously evaluate the adverse effects of antidepressants on fish, it is necessary to clarify their specific interactions with fish MATs based on the mode of action (MoA).

We previously demonstrated that zebrafish possess orthologs of human MAT genes and the transporters exhibit inhibitory responses to pharmaceuticals in vitro;^25,26^ however, little is known about MAT drug sensitivity in other fish lineages. Research encompassing a broader range of fish species is necessary to better establish the potential for widespread ecosystem effects of environmental pharmaceutical contamination. To address this knowledge gaps, in this study we set out to elucidate the molecular characteristics of fish MATs as potential targets for antidepressants in two phylogenetically distinct teleost species: medaka (*Oryzias latipes*) and ayu (*Plecoglossus altivelis*). Medaka is a small freshwater teleost fish originally inhabiting East Asia but established globally as laboratory animal model for ecotoxicological research, and now also used as a model in neuroscience for studying social and reproductive behaviors.^27–29^ The Ayu is widely distributed in East Asia and commercially important for inland fisheries. Phylogenetically, the ayu occupies an important position within the order Osmeriformes.^30^ It is a semelparous and diadromous fish, migrating between the sea and upstream rivers, with a repertoire of fascinating behaviors of great interest in behavioral ecology.^31^ Wild diadromous fish are directly exposed to wastewater effluents during their upstream migration, and as such evaluating the molecular sensitivity of ayu MATs provides a highly realistic, environmentally relevant link to ecosystem health and fisheries management. Current ecotoxicological data are heavily biased toward a few standard laboratory model species, such as zebrafish and it is critical to determine whether the pharmaceutical sensitivity is restricted to specific laboratory models or a generalizable physiological trait conserved across diverse teleost species. In this study, we characterize MAT responses in medaka and ayu for the first time, demonstrating cross-species patterns and differences. We cloned genes encoding MATs (SERTs, DAT and NET) from both medaka and ayu, and characterized their transport activity and sensitivity to several pharmaceuticals using in vitro assays. This approach allowed us to make a direct comparison of MAT functions across these two divergent species, thereby establishing a more generalized and ecologically realistic framework for fish MAT sensitivities. Our results advance understanding of the mechanistic basis of pharmaceutical pollution and provide evidence of phylogenetic differences in the responsiveness of fish MATs to neuroactive environmental contaminants.

## Materials and Methods

### Animals

The inbred medaka strain, Cab (*O. latipes*), was supplied from National Institute for Basic Biology, Japan. The fish were kept at 26 ± 2 °C under photoperiodic conditions of 14 h light:10 h dark and fed dry powder feed (Hikari; Meito Suien, Nagoya, Japan). Ayu (*P. altivelis*) farmed at the Nagasaki Prefectural Institute of Fisheries (Nagasaki, Japan) were transported to Institute for East China Sea Research, Nagasaki University, Japan, where they were sacrificed. All procedures and protocols were approved by the Institutional Animal Care and Use Committee at Nagasaki University and Tokyo University of Science (K22013).

### Cloning and RT-PCR, Phylogenetic Analysis

The full-coding region of medaka and ayu MAT genes were amplified by standard RT-PCR procedures. Total RNA was isolated from brain tissue from mature animals using the ISOGEN II reagent (Nippongene, Tokyo, Japan) and reverse transcribed with PrimeScript RT reagent Kit (Takara, Kusatsu, Japan) or SuperScript III Reverse Transcriptase (Thermo Fisher Scientific, Waltham, MA, USA) as described in the manufacture’s protocol. Predicted sequences were obtained from Genbank (medaka) and a database built by our RNA sequence analysis (Ayu; transcriptome analysis will be published as another study). Primers were designed to the 5′- and 3′-untranslated regions when possible (Supporting Table S1). They were then subcloned into the mammalian expression vector pcDNA3.1 (Thermo Fisher Scientific). Human (*Homo sapiens*) and zebrafish (*Danio rerio*) cDNAs and expression vectors are described elsewhere.^25,26^ Throughout this manuscript, species-specific prefixes are used for transpoeter genes and proteins: ol for medaka (*O. latipes*), pa for ayu (*P. altivelis*), dr for zebrafish (*D. rerio*), and hs for human (*H. sapiens*).

The deduced amino acid sequences were aligned using the MUSCLE program.^32^ Alignments and questionable gaps were removed. A maximum likelihood tree based on the JTT-matrix-based model was constructed from this alignment using a 1000 replicate bootstrap analysis using MEGA 11 software.^33,34^ The accession numbers of the sequences used in the phylogenetic analyses are listed in Table S2.

To evaluate the tissue-specific expression of olSERTa and olSERTb, total RNA was extracted from (brain, gonads, liver, kidney) of mature medaka and cDNA was synthesized as described above. RT-PCR was performed using gene-specific primers (Table S1) and PCR products were resolved on 1.5% agarose gels. Medaka elongation factor 1a (EF1a) was amplified as an internal control.

### In Situ Hybridization for Gene Expression Analysis

Whole brain from male medaka fixed in paraformaldehyde (PFA; Sigma-Aldrich, St. Louis, MO, USA) were embedded in paraffin. Detailed experimental procedures for in situ hybridization are provided in the Supporting Methods S1. The preparation of the digoxigenin-labeled probes was performed according to the manufacturer’s instructions (Roche, Mannheim, Germany). The templates were obtained by standard RT-PCR procedures and the primer sequences are listed in Table S1.

### In Vitro MAT Inhibition Assay

The pharmaceuticals tested as MAT-targeting antidepressants (or those potentially targeting MATs^26^) were purchased with high chemical purity (>98%) and are listed in Table S3. Since it was impractical to test all commercially available antidepressants, we selected representative compounds based on their high consumption rates, utilizing domestic consumption statistics from Japan and the United Kingdom.^26^ These chemicals were dissolved in Milli-Q water or dimethyl sulfoxide (DMSO; Fujifilm Wako Pure Chemical, Osaka, Japan). The final concentration of DMSO in the assay did not exceed 0.1% to avoid cytotoxicity.

The in vitro MAT inhibition assay is based on the active uptake of the fluorescent substrate 4-(4-Dimethylamino)­phenyl-1-methylpyridinium iodide (APP, also known as IDT307; Sigma-Aldrich) into cells via exogenously expressed transporter proteins.^25,26^ The accumulated intracellular APP is then quantified by measuring its fluorescence intensity; thus, a reduction in fluorescent signal indicates concentration-dependent inhibition of transporter function by the tested antidepressants. Plasmids encoding MATs were transiently transfected with the Lipofectamine 2000 reagent (Thermo Fisher Scientific) into HEK-293 cells (DS Pharma Biomedical, Osaka, Japan). Transfection efficiencies or protein expression levels were confirmed that the baseline fluorescence intensity of accumulated APP was equivalent in cells subjected to transient transfection across assays. Transfected cells were then exposed to a dilution series of antidepressants for 10 min. Cells were exposed to each chemical ranging from 10^–10^ M to 10^–5^ M using 10-fold serial dilutions. The maximum chemical concentration (10^–5^ M) and the solvent concentration (0.1% DMSO) were determined based on previously validated protocols to ensure that no cytotoxicity or compromise in cell viability occurred in the HEK-293 cells during the assay period.^25,26^ All chemical solutions were visually inspected prior to the assay to ensure complete solubility at the highest concentration tested. After addition of APP (1 μM), cells were incubated in a 37 °C for 1 h. APP fluorescence inside cells was measured using a microplate reader (FilterMax F5; Molecular Devices, San Jose, CA, USA). All assays were performed at least in triplicate for each antidepressant (*n* ≥ 3). APP uptake was normalized to the maximum APP fluorescence (i.e., APP uptake relative to maximum %). To derive IC_50_ values, dose–response curves were fitted using a three-parameter nonlinear regression model with a constrained Hill slope (Hill slope = −1.0) using GraphPad Prism 5 software (GraphPad Software, La Jolla, CA, USA).

## Results

### Isolation of Monoamine Transporters from Medaka and Ayu

We identified four putative MAT genes in the medaka (*O. latipes*, Hd-rR strain) from the GenBank database: two *O. latipes* SERTs (olSERTa and two alternative splicing forms of SERTb), *O. latipes* NET (olNET) and *O. latipes* DAT (olDAT) (Table S2). Based on these reference sequences, we successfully amplified the full-length cDNAs of these MATs from medaka (Cab strain) adult brain cDNA as a template via RT-PCR. Comparison of our isolated cDNA sequences with the reference sequences revealed high identity, except for one amino acid substitution in olSERTa (glutamate to glycine at position 18; E18G) and four substitutions in olNET (A50P, E236Q, V250L, and H604C). The two olSERTb splice variants, hereafter referred to as olSERTbx1 and olSERTbx2, differed in their N-terminal regions (Figure S1A). The N-terminus of olSERTbx1 showed higher similarity to that of the zebrafish (*D. rerio*) SERTb ortholog (drSERTb), whereas a corresponding sequence for the N-terminally truncated olSERTbx2 was not found in other available teleost databases. Therefore, olSERTbx1 was considered the primary olSERTb ortholog for subsequent analyses. While an N-terminally truncated splice variant of SERTa is registered for zebrafish (XP_009289948), no such variant was found for olSERTa. No splice variants were identified for *olDAT* or *olNET* in our study. Subsequently, we cloned the four corresponding MAT orthologs from ayu (*P. altivelis*): paSERTa (LC843755), paSERTb (LC843756), paNET (LC843757), and paDAT (LC843758). No splice variants for these ayu MATs were identified in the current study.

We next evaluated the conservation of key amino acid residues required for transporter function in mammals. MATs facilitate monoamine reuptake by utilizing sodium and chloride ion gradients.^35^ Residues predicted to form the binding sites for sodium and chloride ions were highly conserved between human and fish MATs; however, olSERTbx2 lacked several residues in the sodium binding sites (Figure S2A–C). Similarly, most residues predicted to interact with endogenous substrates (i.e., 5-HT for SERT, norepinephrine for NET, and dopamine for DAT, respectively) were also well conserved (Figure S2A–C). Intriguingly, we identified several substitutions in functionally important regions. For instance, residues Y95, I172 and A173 in human (*H. Sapiens*) SERT (hsSERT) were conserved in the fish SERTa orthologs, but were substituted with F, V and S, respectively, in the SERTb orthologs (Figure S2A). Furthermore, a key phenylalanine residue in hsNET (F317) and hsDAT (F155) was consistently replaced by a tyrosine in the corresponding medaka and ayu orthologs (Figure S2B, C).

A molecular phylogenetic analysis of MATs from medaka, ayu, zebrafish, and human revealed that they clustered into three distinct clades corresponding to SERT, NET and DAT (Figure 1). The teleost SERT sequences further segregated into two distinct subclades, SERTa and SERTb. Notably, human SERT clustered within the teleost SERTa subclade. A broader phylogenetic analysis including other chordates suggested that the duplication event of SERTa and SERTb paralogs occurred early in the vertebrate lineage, and that the SERTb paralog was subsequently lost in the mammalian lineage (Figure S3).

**Figure 1:**
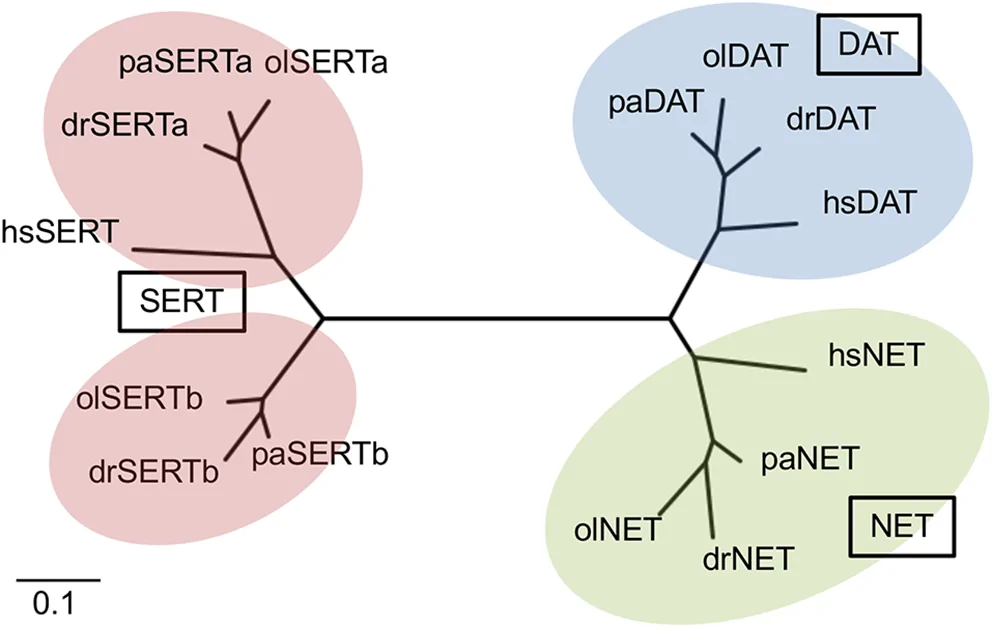
The evolutionary relationship of medaka, ayu, zebrafish, and human monoamine transporters. A maximum likelihood tree was constructed using a 1000 replicate bootstrap analysis and the tree with the highest log likelihood is shown. The scale bar represents 0.1 substitutions per site. The accession number of the sequences used in the tree are listed in the Table S2. Prefixes in gene names indicate the species of origin: ol, *O. latipes* (medaka); pa, *P. altivelis* (ayu); dr, *D. rerio* (zebrafish); hs, *H. sapiens* (human).

RT-PCR analysis revealed that two paralogs displayed distinct tissue localization patterns. olSERTa was expressed in the brain but also widely expressed peripheral tissues. In contrast, olSERTb transcript expression was restricted to the brain, with a very faint band detected in the ovary (Figure S4).

### Expression Pattern of Monoamine Transporter Genes in Medaka Brain

We then investigated the localization of MATs in the adult medaka brain using in situ hybridization. Each MAT transcript exhibited a distinct expression pattern within specific regions of the brain. Specifically, SERTa was expressed in the nucleus dorsomedialis thalami (DM), nucleus interpeduncularis (NIP), and fasciculus longitudinalis medialis (flm) (Figure 2A–C). In contrast, no clear signals for SERTb were detected in the medaka brain (data not shown). NET was expressed in the torus longitudinalis (TL), locus coeruleus (LC), and nucleus motorius nervi vagi (NXm) (Figure 2D–F). DAT was expressed in the nucleus tractus mesencephalicus nervi trigemini (NTMT), nucleus diffuses lobi inferioris (NDLI) and flm (Figure 2G,H).

**Figure 2:**
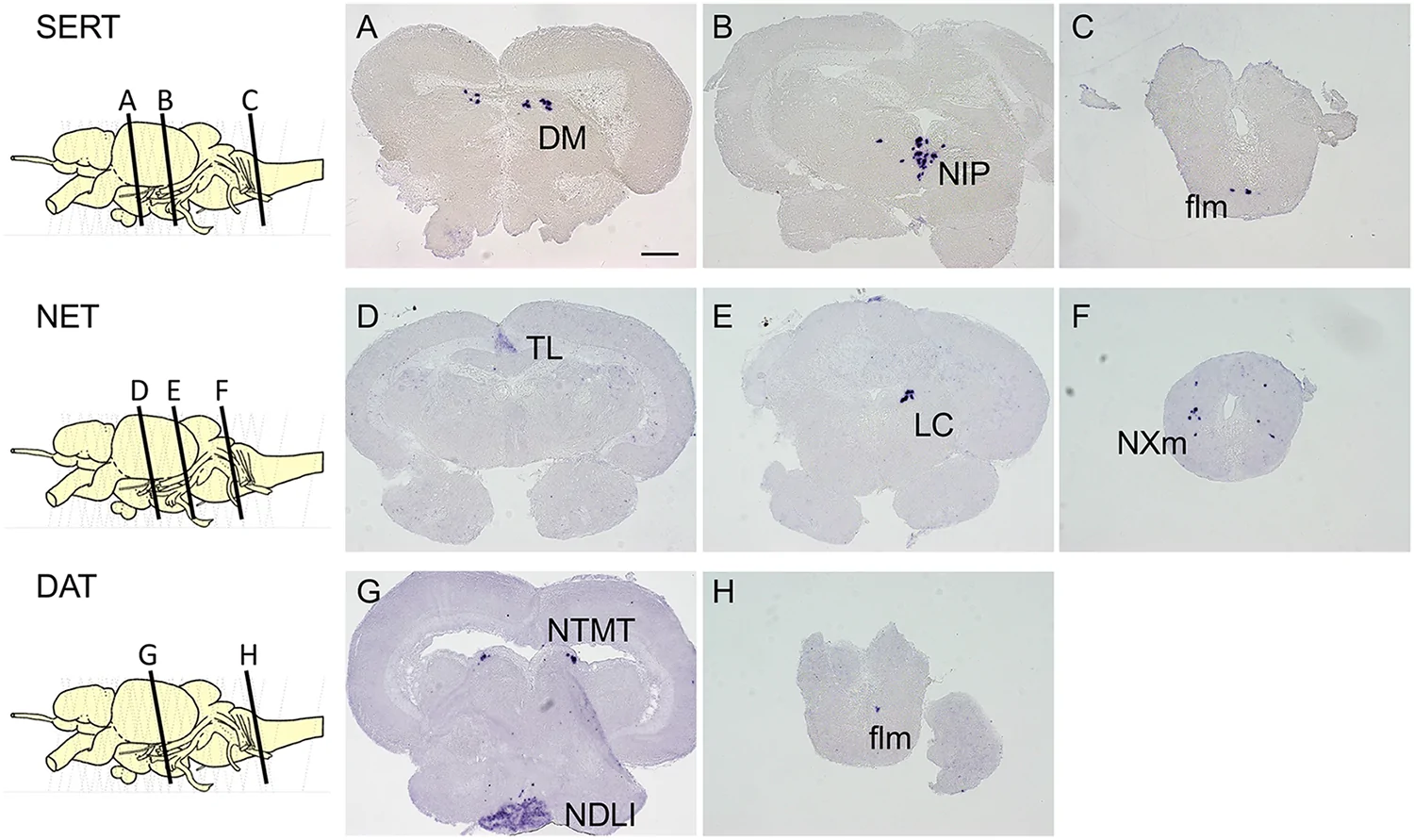
Expression pattern of monoamine transporter genes in medaka brain. The expression of SERTa, DAT, and NET in the medaka brain at the section indicated in the schematic diagram is shown by in situ hybridization analysis. DM, dorsomedialis thalami; FLM, fasciculus longitudinalis medialis; LC, locus coeruleus; NDLI, nucleus diffuses lobi inferioris; NIP, nucleus interpeduncularis; NTMT, nucleus tractus mesencephalicus nervi trigemini; NXm, nucleus motorius nervi vagi; TL, torus longitudinalis.

### Activity of Antidepressants against Medaka and Ayu Monoamine Transporters

To characterize the pharmacological properties of the cloned medaka MATs, we measured their inhibition for exposure to several antidepressants using a cell-based fluorescent substrate (APP) uptake assay.^25^ First, we confirmed that cells expressing olSERTa, olSERTb, olNET, or olDAT exhibited robust APP uptake, whereas cells expressing the truncated olSERTbx2 did not (Figure S4). Consequently, the olSERTbx2 was excluded from subsequent inhibition assays. Scale bar = 200 μm.

We then measured the inhibitory effects of representative antidepressants on olSERTa, olSERTb, olNET, and olDAT: sertraline [known as SSRI and dopamine reuptake inhibitor (DRI)], fluoxetine (SSRI), clomipramine (TCA), duloxetine (SNRI), bupropion (DRI) (Figure 3). Overall, all tested medaka MATs were inhibited by these antidepressants, except for bupropion, which selectively inhibited olDAT, consistent with its primary action as a DRI. These response profiles are generally consistent with the known specificities of these antidepressants for human MATs. Among these pharmaceuticals (except bupropion), olSERTa was consistently inhibited at lower concentrations compared with olSERTb and therefore, only SERTa was used in subsequent SERT assays. Expanded data regarding the IC_50_ values of antidepressants for medaka MATs are shown in Figure S6 and Table 1. In the current assay, while most pharmaceuticals exhibited the strongest effects on olSERTa, norquetiapine, atomoxetine, desipramine, mianserin and maprotiline were more potent in their inhibition of olNET (Figure 4). Although bupropion specifically inhibited DAT, the IC_50_ values for sertraline, duloxetine, and atomoxetine against olDAT were lower than those for bupropion, despite their lack of specificity for olDAT (Figure 4).

**Figure 3:**
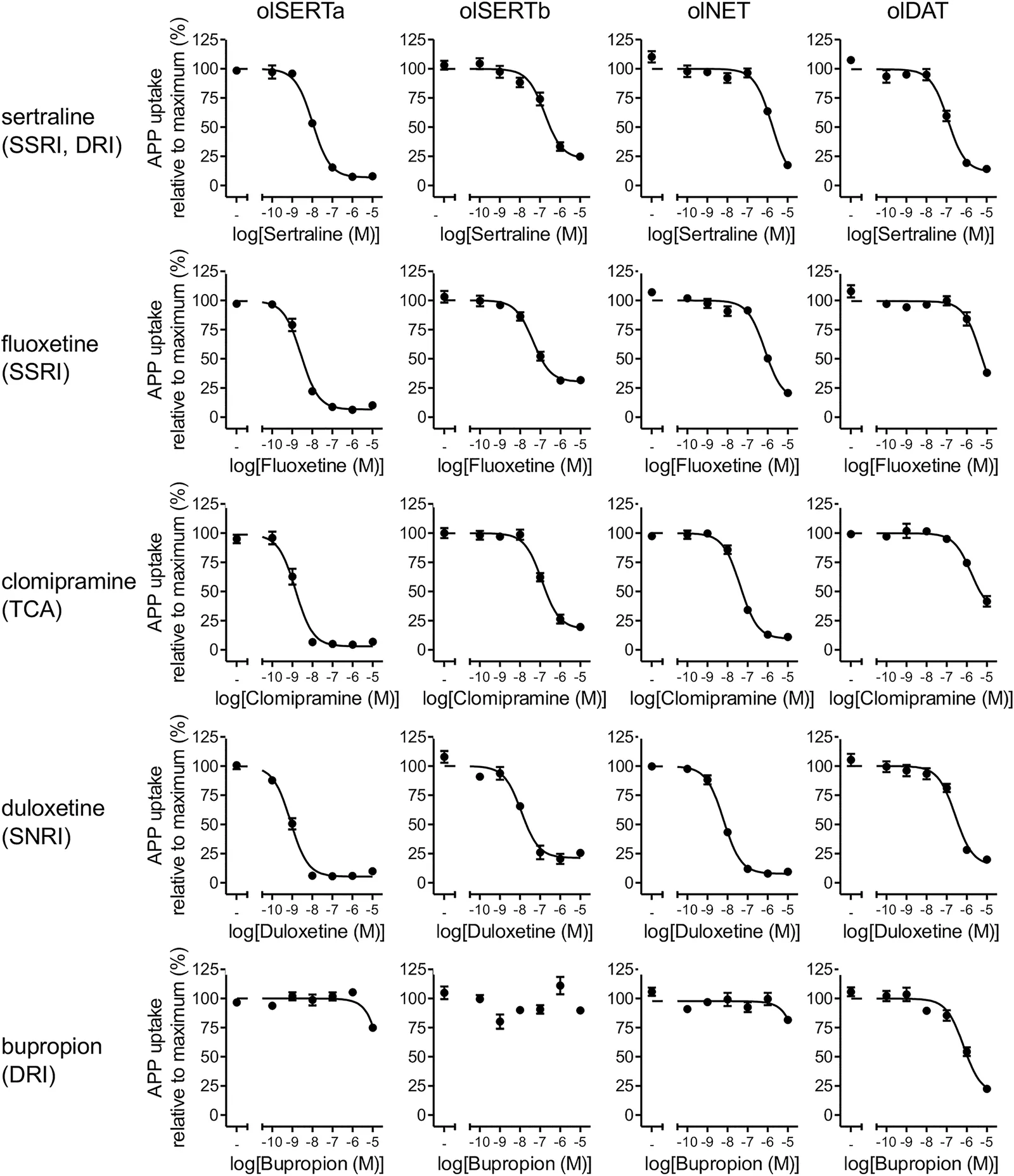
APP uptake inhibition by pharmaceutical antidepressants for medaka monoamine transporters. Effect of antidepressants (Sertraline, Fluoxetine, Clomipramine, Duloxetine, and Bupropion) on the inhibition of APP uptake is evaluated as APP fluorescence inside cells (APP uptake relative to maximum, %) for the *O. latipes* SERTa (olSERTa), olNET, and olDAT. Error bars indicate SEM.

**Figure 4:**
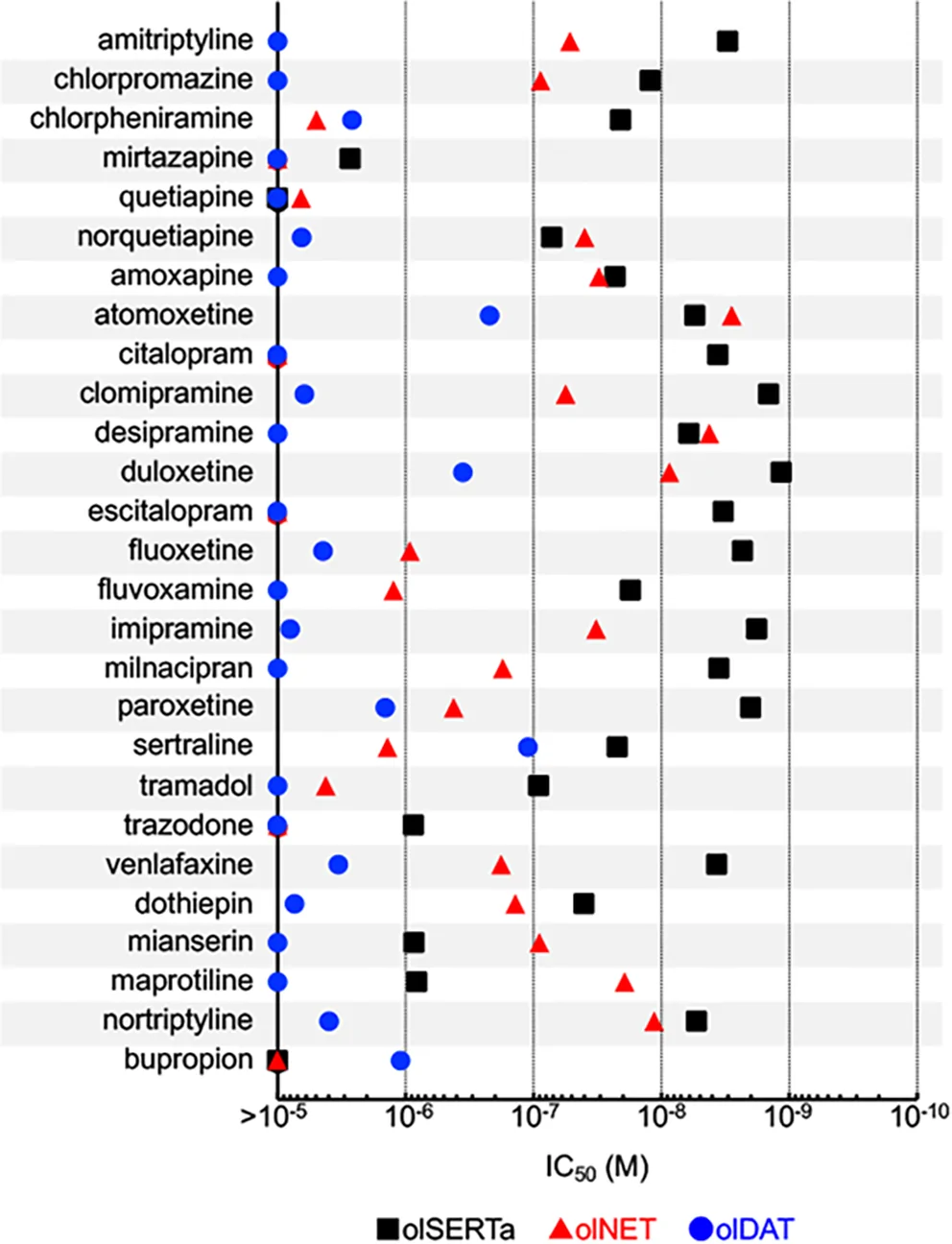
Comparative IC_50_ values of antidepressants against medaka MATs. Plots indicate IC_50_ values (M) of antidepressants measured in this study. Each transporter is represented by a different symbol: *O. latipes* SERTa (olSERTa), black square; olNET, red triangle; and olDAT, blue circle.

**Table 1:**
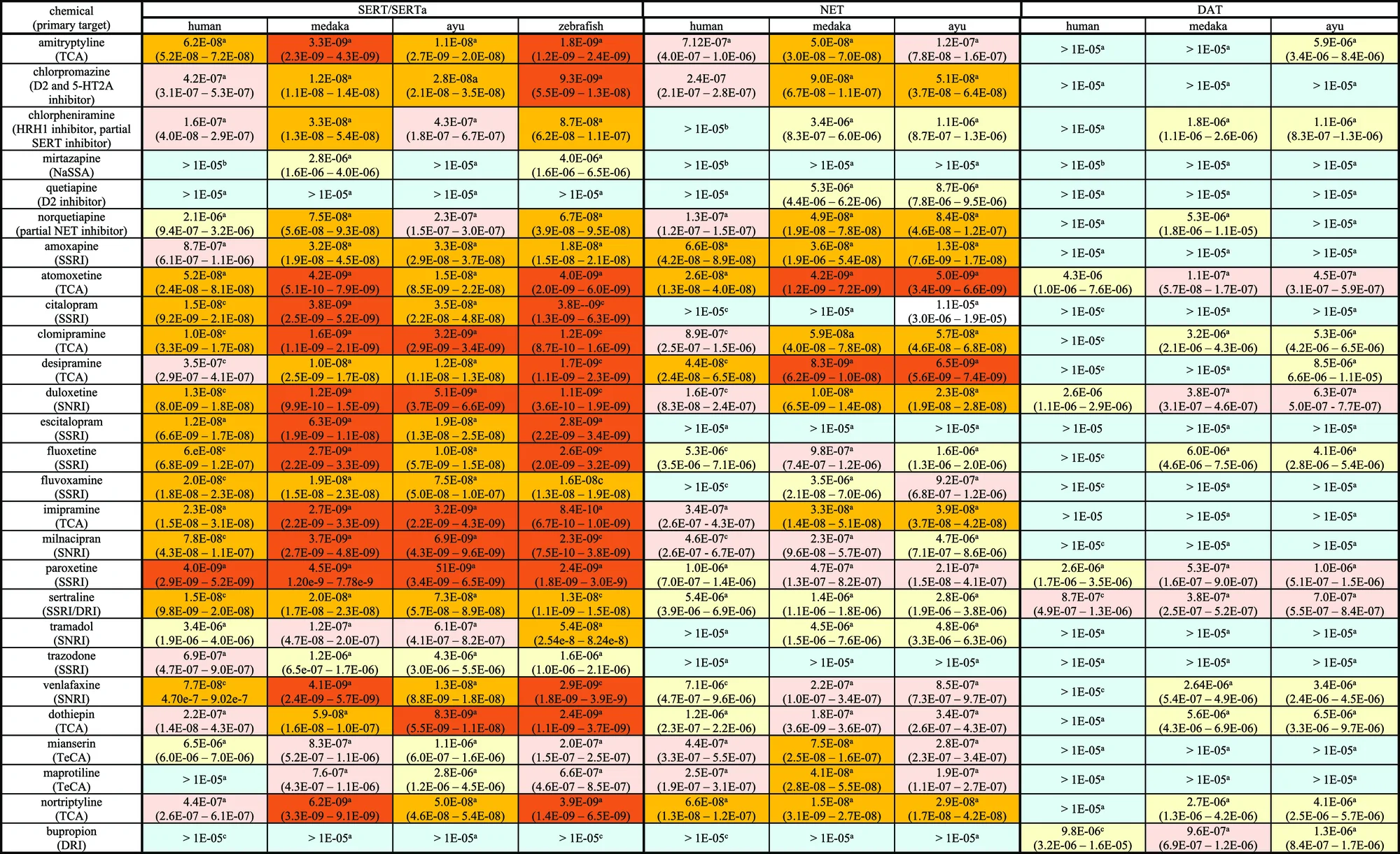
IC_50_ Values and 95% Confidence Intervals of Antidepressants for Human, Madaka, Ayu and Zebrafish MATs^a^

a IC_50_s (M) and 95% confidence intervals (95% CIs (M), in parentheses). The alphabetical symbols indicate the source of the data (a, in this study; b, Zhamg et al., 2023; c, Ihara et al., 2021). D2, dopaine D2 receptor; DRI, dopamine reuptake inhibitor; HRH1, histamine H1 receptor; 5HT-2A, Serotonin 5-HT2A receptor; NaSSA, noradrenergic and specific serotonergic antidepressant; SNRI, serotonin-norepinephrine reuptake inhibitor; SSRI, selective serotonin reuptake inhibitor; TCA, tricyclic antidepressant; TeCA, tetracyclic antidepressants.

We extended pharmacological profiling to the ayu MATs and observed a similar pattern: paSERTa was consistently more sensitive to these pharmaceuticals than paNET and paDAT (Figures S6, S7 and Table 1). Furthermore, in addition to norquetiapine, atomoxetine, desipramine, mianserin and maprotiline, amoxapine and nortriptyline inhibited paNET more strongly than paSERT.

### Comparison of Inhibitory Effect of Pharmaceuticals for Human and Fish SERTs

Given its higher sensitivity to pharmaceutical antidepressants, we focused on SERT for further comparative analyses. We directly compared the inhibitory profiles of the antidepressants across SERT orthologs from medaka, ayu, zebrafish, and human, using data for the latter two species from our previous work.^25,26^ We observed some variations in sensitivity among the fish species, but the overall inhibitory effects for most of the tested antidepressants on medaka and zebrafish SERTa were generally similar (Figure 5). In addition, most tested antidepressants inhibited both hsSERT and the fish SERTa. While paroxetine was the most potent inhibitor of hsSERT, duloxetine was the most effective for medaka and ayu SERTa, alongside clomipramine and imipramine, which were equally potent against ayu SERTa. Remarkably, the fish SERTa orthologs were consistently more sensitive to inhibition than hsSERT, as indicated by their consistently lower IC_50_ values. For some antidepressants, the IC_50_ values for the fish SERTa orthologs were more than 10 times lower than those for hsSERT. For example, the IC50 of desipramine for hsSERT was 350 nM, whereas those for medaka, ayu, and zebrafish SERTa were 6.1 nM, 12 nM and 1.7 nM, respectively. Similarly, amoxapine inhibited fish SERTa at concentrations approximately 23- and 35-fold lower, respectively, than those required to inhibit hsSERT [760 nM vs 23 nM (medaka), 33 nM (ayu) and 22 nM (zebrafish)]. This trend was also observed in the interspecies comparisons of NET and DAT (Figures S8, S9).

**Figure 5:**
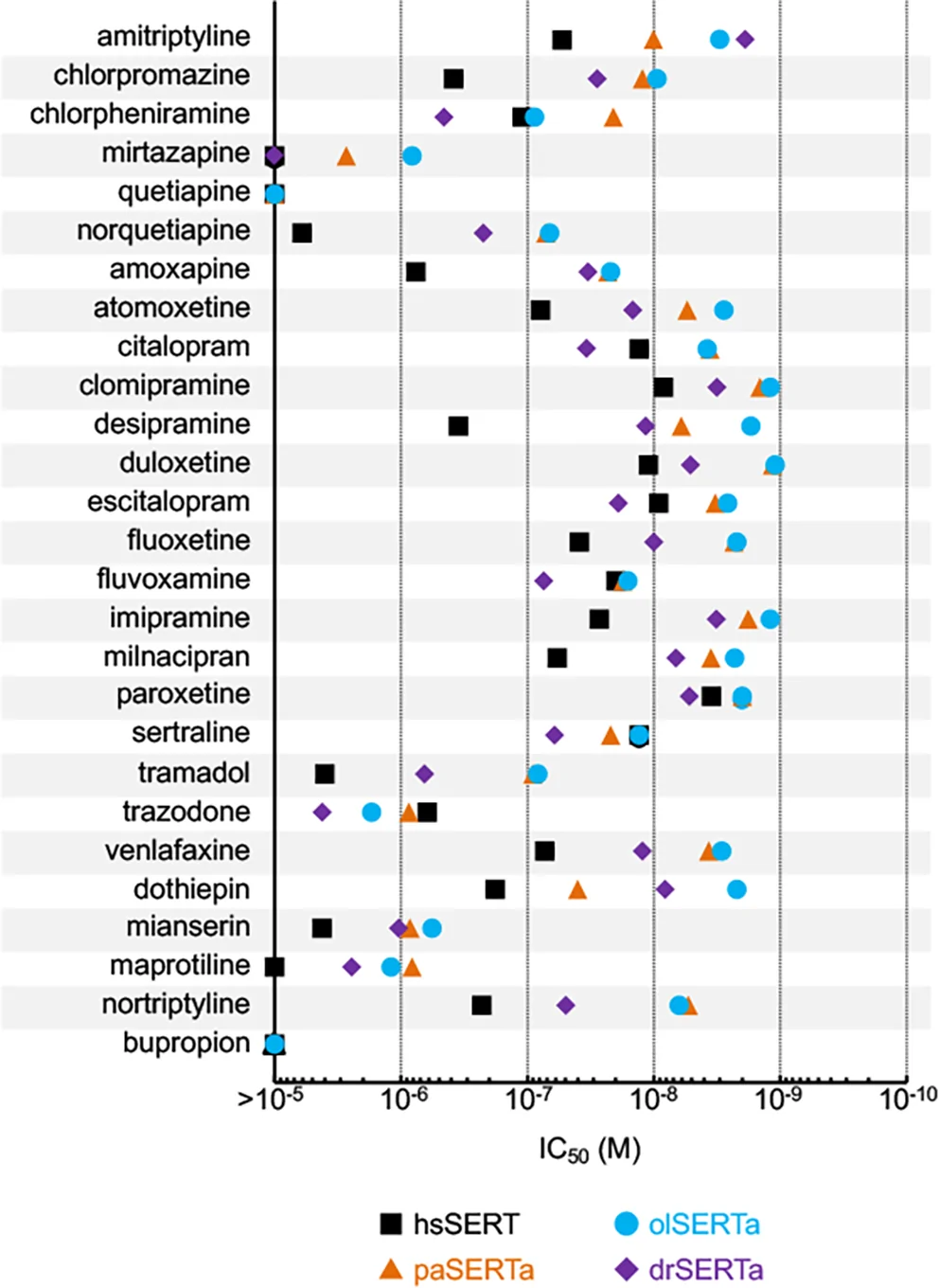
Comparative IC_50_ values of antidepressants against hsSERT, olSERTa, paSERTa, and drSERTa. Plots indicate IC_50_ values (M) of antidepressants with detailed values and 95% confidence intervals provided in Table 1. Each transporter is represented by a different symbol: hsSERT, black square, olSERTa, blue circle; paSERTa, orange triangle; and drSERTa, purple diamond. Prefixes denote species: hs, *H. sapiens* *;* ol, *O. latipes*; pa, *P. altivelis*; dr, *D. rerio*.

Furthermore, some antidepressants exhibited interspecies variations in their relative inhibitory potencies for SERT versus NET. For instance, in humans, norquetiapine is known to act as a highly selective NET inhibitor with a substantially lower potency against SERT.^36^ However, this target selectivity was notably absent in both medaka and ayu, where norquetiapine inhibited SERT and NET with highly comparable potencies.

In the current assay, inhibitory effects were detected even for pharmaceuticals for which MATs were not previously recognized as primary targets (Figure 5 and Table 1). Mirtazapine, a noradrenergic and specific serotonergic antidepressant (NaSSA), is considered to have low affinity for monoamine transporters and indeed did not inhibit human MATs; however, a weak inhibitory effect was observed against olSERT and drSERT. Although quetiapine is primarily known as an inhibitor for human dopamine (D2), serotonin (5-HT2A) and histamine (H1) receptors,^37^ we found that it inhibited olNET and paNET, despite having no effect on hsMATs (Figure S6). Chlorpromazine, which has been reported to interact with human SERT and NET,^38^ affected both human and fish MATs.

## Discussion

In this study, we investigated fish MATs, the primary molecular targets of widely prescribed antidepressants that are found as environmental contaminants globally in surface waters. We first identified and characterized the molecular and evolutionary features of MATs from two phylogenetically distinct teleost fish, the medaka and Ayu. We then performed a comparative pharmacological analysis, revealing that fish SERTa exhibits markedly higher in vitro sensitivity to several antidepressants than has been established for its human ortholog. Collectively, our findings present a robust molecular approach for use in assessing the ecological risks of pharmaceutical pollution and for understanding the mechanisms of their impact on aquatic organisms.

### Evolution of MAT Genes

Although MAT genes emerged early in the bilaterian lineage,^21,24^ their evolution within vertebrates remains insufficiently defined. Our phylogenetic analysis clarifies the evolutionary trajectory of vertebrate MATs, which divide into SERT and catecholamine transporter (NET and DAT) groups. Basal chordates and early vertebrates exhibit varying degrees of MAT conservation and secondary gene loss. For instance, ascidian species only SERT-like sequences, whereas lamprey retain two SERTs, a NET-like and a DAT-like gene.

The timing of the divergence of SERTa and SERTb/SERT-like remains somewhat obscure, however, distinct SERTa and SERTb orthologs are first clearly identifiable in cartilaginous fishes (Figure S3). Lampreys possess two SERT genes (SERT-like1 and SERT-like2) that both cluster with the SERTb clade (Figure S3), suggesting that the lamprey SERTa paralog was secondarily lost in this lineage. Following these early divergence events, most vertebrates have retained both SERTa and SERTb, however, the SERTb paralog was lost early in the mammalian lineage. A similar pattern of gene loss is seen for DAT in birds and reptiles, likely due to functional compensation by the pharmacologically similar NET.^23^ Overlapping substrate specificity among MATs may generally increase the likelihood of gene loss and reorganization within the MAT gene family.

The functional differentiation between SERTa and SERTb/SERT-like has been largely unknown until now. As demonstrated in our pharmacological assay, fish SERTa is similar in responsiveness to human SERT and differing from that of SERTb. Our results provide a compelling link between the evolutionary divergence of these paralogs and their pharmacological properties. We demonstrated that fish SERTa, the paralog retained in the human lineage, is more sensitive to human-targeted antidepressants than is fish SERTb. Our sequence analysis revealed that key residues for antidepressant binding in human SERT [e.g., Y95, I172, A173 ^39^] are conserved in fish SERTa, but are substituted in fish SERTb (e.g., Y95F, I172 V, A173S). The substitution of these critical residues in the SERTb paralog likely underlies its lower sensitivity to these pharmaceuticals, providing a clear mechanistic explanation for the functional differentiation between them.

Tissue localization analysis revealed that olSERTb is strictly restricted to the brain, while olSERTa is widely distributed across both central and peripheral organs. This expression pattern is highly consistent with that reported for zebrafish, where drSERTb is predominantly expressed in the brain.^40^ Despite its brain-specific expression, olSERTb transcripts were not detected via in situ hybridization in the adult medaka brain, which is likely due to its low abundance. This is supported by our transcriptomic data where olSERTb reads account for only 10–15% of olSERTa reads in the adult brain (conducted as part of a separate study). Although olSERTb serves as a low-abundance paralog with lower sensitivity to antidepressants, its evolutionary conservation suggests a distinct physiological role. In zebrafish, SERTa and SERTb are complementarily expressed throughout development,^41^ and SERTa expression levels in offspring are closely linked to behavioral anxiety levels following chemical (atrazine) exposure.^40^ Most notably, SERTa knockout in zebrafish males significantly increases aggression behavior, whereas SERTb knockout leads to decreased aggression.^42^ This contrasting behavioral phenotype provides direct genetic evidence that SERTa and SERTb have evolved distinct, nonoverlapping functions. Future functional studies, such as knockout of olSERTa and olSERTb in medaka, will be invaluable to dissect their discrete pathways and functions of these duplicated transporters throughout teleosts.

### Localization of MATs in the Medaka Brain

The diverse evolution of MAT genes, driven in part by the teleost-specific whole-genome duplication, has yielded multiple paralogs that are often expressed in partially overlapping yet distinct brain regions. Indeed, previous studies have shown that MAT genes in teleost fish exhibit distinct localization patterns that vary among species and gene families.^41,43–45^ For example, two SERT paralogs in zebrafish (drSERTa and drSERTb) are both concentrated in classical serotonergic regions but also show unique expression domains during embryonic stage.^41,43^

Our findings in medaka both align with and extend these observations. While we did not detect a clear signal for olSERTb in the adult medaka brain, we observed distinct expression of olSERTa in the NIP, DM, and flm. This pattern is partially conserved, as drSERTa expression is also found in the raphe nucleus adjacent to the NIP in zebrafish.^41^ In zebrafish, the NIP is implicated in aggressive behavior,^46^ while the DM is a key region for emotion processing, and has been reported as essential for fear responses.^47^ Therefore, it is plausible that these olSERTa-expressing regions in medaka are also involved in modulating emotions and affective states. The action of pharmaceuticals on these specific circuits could thus disrupt behaviors and physiological functions related to anxiety and fear.

Regarding the catecholamine transporters, we detected distinct signals for olDAT in parts of the midbrain, including the NTMT, and in the hypothalamus, particularly the NDLI. The inferior lobe of the teleost hypothalamus is homologous to parts of the mammalian hypothalamus and serves as a key integration center for diverse sensory inputs. This hypothalamic expression of DAT is also conserved in zebrafish,^48^ suggesting a conserved role in modulating responses to sensory stimuli.

Finally, our detection of olNET transcripts in the LC is consistent with a previous report in medaka.^45^ The LC is a highly conserved brainstem nucleus expressing high levels of NET and regulating arousal, attention, and stress responses across vertebrates.^49^ Thus, the LC likely serves as a key site where NET-targeting pharmaceuticals alter fish behaviors.

### Biological Activities of Fish MATs to Antidepressants

The in vitro MAT inhibition assay using the fluorescent substrate APP, originally developed for human (*H. sapiens*) SERT (hsSERT),^50^ has proven to be a valuable tool for comparing the biological activity of MAT inhibitors across both human and fish orthologs.^25,26^ In the present study, we utilized this assay to evaluate the effects of several pharmaceutical compounds on medaka and ayu MATs. Our results show that the pharmacological profiles of antidepressants on medaka and ayu MATs were generally consistent with their known properties on human orthologs. For example, the potent inhibition of SERT and NET by the SNRI duloxetine, and the selective inhibition of DAT by the DRI bupropion, align with the expected target specificities of these pharmaceuticals. However, we also observed that certain compounds, such as mirtazapine and maprotiline, inhibited APP uptake in fish SERTa despite showing no inhibitory effect on hsSERT. Similar discrepancies were found for other transporters; for instance, fluvoxamine for NET, and venlafaxine and dothiepin for DAT. We suggest therefore, these pharmaceuticals may trigger unintended effects in fish through pathways (i.e., MATs) that differ from the anticipated modes of action in humans.

Consistent with our phylogenetic analysis, which clusters human SERT within the SERTa clade, SERTa is markedly more sensitive to these neuroactive pharmaceuticals than SERTb. Focusing on SERTa, our results reveal that although these pharmaceuticals were designed for human MATs, they are highly effective against fish orthologs. Our previous work showed that the IC_50_ values for most antidepressants against zebrafish SERTa were lower than those against human SERT.^26^ Our current results further corroborate this trend, demonstrating that this high sensitivity is a consistent feature across diverse fish SERTa orthologs. By contrast, sensitivity to some pharmaceuticals varied among the fish species. Although ayu and zebrafish SERTa are phylogenetically closer in amino acid sequence, the pharmacological responses of medaka and zebrafish SERTa were more similar for most tested antidepressants, contrasting with the clear phylogenetic trends reported for estrogen receptors.^51,52^ In the future, determining the critical amino acid residues for antidepressant binding through molecular docking and structural modeling may provide a mechanistic explanation for these interspecies differences in reactivity.

The molecular and pharmacological data set obtained in this study provides a valuable resource for the environmental risk assessment of antidepressants. By demonstrating that key molecular targets in fish can be more sensitive than their human orthologs, our work offers crucial insights into the potential risks of pharmaceutical exposure for diverse aquatic wildlife, here for fish. To assess the environmental relevance of our in vitro findings, we compared our measured IC_50_ values with actual concentration ranges detected in aquatic environments. Antidepressants are frequently detected in wastewater effluents and surface waters at concentrations ranging from low ng/L to several μg/L.^5,6^ Notably, the IC_50_ values determined in this study for several potent inhibitors of fish SERTa, such as duloxetine (1.2 nM ≈ 357 ng/L for medaka SERTa), fluoxetine (2.7 nM ≈ 835 ng/L), citalopram (3.8 nM ≈ 1,233 ng/L), and paroxetine (4.5 nM ≈ 1,334 ng/L), directly overlap with the higher-end concentrations reported in heavily impacted aquatic systems.^8^ Furthermore, while our assay focused on single compounds, it is important to recognize that pharmaceuticals exist as complex mixtures in the environment, and given that many neuroactive compounds in the environment that share common target sites there are likely additive effects on these molecular targets.^25,26^ It is therefore plausible that these neuroactive pharmaceuticals, even when present in the environment at levels that are insufficient individually to perturb MATs function can (and likely) do so in fish for the known environmental mixtures.

This molecular level vulnerability directly aligns with observed in vivo behavioral and physiological perturbations in teleosts. For example, previous in vivo exposure studies have demonstrated that environmentally realistic concentrations of SSRIs (e.g., 100 ng/L to 10 μg/L of fluoxetine) induce behavioral alterations in fish.^19,53,54^ We demonstrated that olSERTa is expressed in the NIP and DM, where the NIP is closely associated with aggressive behavior and social status,^46^ and the DM serves as a homologous structure to the mammalian amygdala, playing a pivotal role in mediating fear and anxiety responses.^47^ The striking alignment between our in vitro IC_50_ values and the effective concentration ranges that trigger in vivo behavioral abnormalities suggests that the direct inhibition of SERTa in these specific emotional-processing brain circuits represents a key Molecular Initiating Event (MIE) in the adverse outcome pathway (AOP) of pharmaceutical pollution in fish. To address this, future in vivo studies are necessary to evaluate environmentally realistic exposure routes and internal pharmacokinetics, including the absorption, tissue distribution, transport, and metabolism of these compounds.^55^ The comparative framework established here provides a powerful tool for dissecting complex interactions and better predicting the ecological consequences of pharmaceutical pollution. Taken together, our findings demonstrate that relying solely on mammalian data for chemical effect measures can result in severe considerable underestimations of their ecotoxicological risks to aquatic systems. By identifying fish-specific sensitivities, this research provides a vital scientific basis for prioritizing target pharmaceuticals in environmental monitoring programs and for deriving more protective, species-specific risk thresholds in water quality guidelines. Incorporating these molecular and MoA-based sensitivity profiles into environmental policy frameworks will enable the implementation of precautionary chemical management strategies to protect aquatic ecosystems.

## Supplementary Material



## Notes

The additional method (primer sequences, accession number of genes, test chemicals) and data (sequence comparison, extended phylogenic tree, tissue distribution of SERT genes, extended data of APP assay, comparison of IC_50_ values of antidepressants against NET and DAT) (PDF)

## References

[1] Goulty M., Botton-Amiot G., Rosato E., Sprecher S. G., Feuda R. (2023). The monoaminergic
system is a bilaterian innovation. Nat. Commun..

[2] Kristensen A. S., Andersen J., Jorgensen T. N., Sorensen L., Eriksen J., Loland C. J., Stromgaard K., Gether U. (2011). SLC6 neurotransmitter
transporters: structure, function, and regulation. Pharmacol. Rev..

[3] Lin L., Yee S. W., Kim R. B., Giacomini K. M. (2015). SLC transporters
as therapeutic targets: emerging opportunities. Nat. Rev. Drug Discovery.

[4] Jarończyk M., Walory J. (2022). Novel molecular targets
of antidepressants. Molecules.

[5] Writer J. H., Ferrer I., Barber L. B., Thurman E. M. (2013). Widespread occurrence
of neuro-active pharmaceuticals and metabolites in 24 minnesota rivers
and wastewaters. Sci. Total Environ..

[6] Schultz M. M., Furlong E. T., Kolpin D. W., Werner S. L., Schoenfuss H. L., Barber L. B., Blazer V. S., Norris D. O., Vajda A. M. (2010). Antidepressant
pharmaceuticals in two U.S. effluent-impacted streams: occurrence
and fate in water and sediment, and selective uptake in fish neural
tissue. Environ. Sci. Technol..

[7] Pivetta R.
C., Rodrigues-Silva C., Ribeiro A. R., Rath S. (2020). Tracking the occurrence
of psychotropic pharmaceuticals in Brazilian wastewater treatment
plants and surface water, with assessment of environmental risks. Sci. Total Environ..

[8] Mole R. A., Brooks B. W. (2019). Global scanning
of selective serotonin reuptake inhibitors:
occurrence, wastewater treatment and hazards in aquatic systems. Environ. Pollut..

[9] González
Alonso S., Catala M., Maroto R. R., Gil J. L., de Miguel A. G., Valcarcel Y. (2010). Pollution by psychoactive pharmaceuticals
in the Rivers of Madrid metropolitan area (Spain). Environ. Int..

[10] Giebułtowicz J., Nalecz-Jawecki G. (2014). Occurrence
of antidepressant residues in the sewage-impacted
Vistula and Utrata rivers and in tap water in Warsaw (Poland). Ecotoxicol. Environ. Saf..

[11] Batt A. L., Kincaid T. M., Kostich M. S., Lazorchak J. M., Olsen A. R. (2016). Evaluating the extent of pharmaceuticals
in surface
waters of the United States using a National-scale Rivers and Streams
Assessment survey. Environ. Toxicol. Chem..

[12] Arnold K.
E., Brown A. R., Ankley G. T., Sumpter J. P. (2014). Medicating the environment:
assessing risks of pharmaceuticals to wildlife and ecosystems. Philos. Trans. R. Soc., B.

[13] Liu Y., Lv J., Guo C., Jin X., Zuo D., Xu J. (2025). Environmental
behavior, risks, and management of antidepressants in the aquatic
environment. Environ. Sci. Process Impacts.

[14] Ramos C., da Silva B. D., Conte-Junior C. A. (2026). Antidepressants
and anxiolytics in
aquatic environments as emerging contaminants and their role in antibiotic
resistance. Sci. Total Environ..

[15] Wong R. Y., Oxendine S. E., Godwin J. (2013). Behavioral
and neurogenomic transcriptome
changes in wild-derived zebrafish with fluoxetine treatment. BMC Genomics.

[16] Margiotta-Casaluci L., Owen S. F., Cumming R. I., de Polo A., Winter M. J., Panter G. H., Rand-Weaver M., Sumpter J. P. (2014). Quantitative cross-species
extrapolation between humans and fish: the case of the anti-depressant
fluoxetine. PLoS One.

[17] Cachat J., Stewart A., Grossman L., Gaikwad S., Kadri F., Chung K. M., Wu N., Wong K., Roy S., Suciu C., Goodspeed J., Elegante M., Bartels B., Elkhayat S., Tien D., Tan J., Denmark A., Gilder T., Kyzar E., Dileo J., Frank K., Chang K., Utterback E., Hart P., Kalueff A. V. (2010). Measuring
behavioral and endocrine responses to novelty stress in adult zebrafish. Nat. Protoc..

[18] Ansai S., Hosokawa H., Maegawa S., Kinoshita M. (2016). Chronic fluoxetine
treatment induces anxiolytic responses and altered social behaviors
in medaka, *Oryzias latipes*. Behav. Brain Res..

[19] Valenti T. W., Gould G. G., Berninger J. P., Connors K. A., Keele N. B., Prosser K. N., Brooks B. W. (2012). Human therapeutic
plasma levels of
the selective serotonin reuptake inhibitor (SSRI) sertraline decrease
serotonin reuptake transporter binding and shelter-seeking behavior
in adult male fathead minnows. Environ. Sci.
Technol..

[20] Kellner M., Olsen K. H. (2020). Divergent response to the SSRI citalopram in male and
female three-spine sticklebacks (*Gasterosteus aculeatus*). Arch. Environ. Contam. Toxicol..

[21] Yamamoto K., Vernier P. (2011). The evolution of dopamine
systems in chordates. Front. Neuroanat..

[22] Mennigen J. A., Stroud P., Zamora J. M., Moon T. W., Trudeau V. L. (2011). Pharmaceuticals
as neuroendocrine disruptors: lessons learned from fish on Prozac. J. Toxicol. Environ. Health B.

[23] Lovell P. V., Kasimi B., Carleton J., Velho T. A., Mello C. V. (2015). Living
without DAT: Loss and compensation of the dopamine transporter gene
in sauropsids (birds and reptiles). Sci. Rep..

[24] Caveney S., Cladman W., Verellen L., Donly C. (2006). Ancestry of neuronal
monoamine transporters in the Metazoa. J. Exp.
Biol..

[25] Ihara M., Zhang H., Ihara M. O., Kato D., Tanaka H. (2021). Proposal for
fluorescence-based in vitro assay using human and zebrafish monoamine
transporters to detect biological activities of antidepressants in
wastewater. Sci. Total Environ..

[26] Zhang H., Kato D., Ihara M. O., Jurgens M. D., Johnson A. C., Chen J., Tanaka H., Ihara M. (2023). Biological-activity-based
prioritization of antidepressants in wastewater in England and Japan. Environ. Sci. Technol..

[27] Yokoi S., Naruse K., Kamei Y., Ansai S., Kinoshita M., Mito M., Iwasaki S., Inoue S., Okuyama T., Nakagawa S., Young L. J., Takeuchi H. (2020). Sexually dimorphic
role of oxytocin in medaka mate choice. Proc.
Natl. Acad. Sci. U.S.A..

[28] Yamashita J., Takeuchi A., Hosono K., Fleming T., Nagahama Y., Okubo K. (2020). Male-predominant galanin
mediates androgen-dependent aggressive chases
in medaka. eLife.

[29] Okuyama T., Yokoi S., Abe H., Isoe Y., Suehiro Y., Imada H., Tanaka M., Kawasaki T., Yuba S., Taniguchi Y., Kamei Y., Okubo K., Shimada A., Naruse K., Takeda H., Oka Y., Kubo T., Takeuchi H. (2014). A neural mechanism underlying mating preferences for
familiar individuals in medaka fish. Science.

[30] Betancur-R R., Wiley E. O., Arratia G., Acero A., Bailly N., Miya M., Lecointre G., Orti G. (2017). Phylogenetic classification
of bony fishes. BMC Evol. Biol..

[31] Tanaka Y., Iguchi K., Yoshimura J., Nakagiri N., Tainaka K. (2011). Historical
effect in the territoriality of ayu fish. J.
Theor. Biol..

[32] Edgar R. C. (2004). MUSCLE:
a multiple sequence alignment method with reduced time and space complexity. BMC Bioinf..

[33] Tamura K., Stecher G., Kumar S. (2021). MEGA11: Molecular evolutionary genetics
analysis version 11. Mol. Biol. Evol..

[34] Stecher G., Tamura K., Kumar S. (2020). Molecular evolutionary genetics analysis
(MEGA) for macOS. Mol. Biol. Evol..

[35] Koldsø H., Christiansen A. B., Sinning S., Schiott B. (2013). Comparative modeling
of the human monoamine transporters: similarities in substrate binding. ACS Chem. Neurosci..

[36] Jensen N. H., Rodriguiz R. M., Caron M. G., Wetsel W. C., Rothman R. B., Roth B. L. (2008). N-desalkylquetiapine,
a potent norepinephrine reuptake
inhibitor and partial 5-HT1A agonist, as a putative mediator of quetiapine’s
antidepressant activity. Neuropsychopharmacology.

[37] Katzung, B. ; Masters, S. ; Trevor, A. Basic and Clinical Pharmacology, 12th ed.; McGraw-Hill Medical, 2012.

[38] Tatsumi M., Jansen K., Blakely R. D., Richelson E. (1999). Pharmacological
profile of neuroleptics at human monoamine transporters. Eur. J. Pharmacol..

[39] Coleman J. A., Gouaux E. (2018). Structural basis for
recognition of diverse antidepressants
by the human serotonin transporter. Nat. Struct.
Mol. Biol..

[40] Lamb S. D., Chia J. H. Z., Johnson S. L. (2020). Paternal exposure
to a common herbicide
alters the behavior and serotonergic system of zebrafish offspring. PLoS One.

[41] Norton W. H., Folchert A., Bally-Cuif L. (2008). Comparative
analysis of serotonin
receptor (HTR1A/HTR1B families) and transporter (slc6a4a/b) gene expression
in the zebrafish brain. J. Comp. Neurol..

[42] Tea M., Pan Y. K., Lister J. G. R., Perry S. F., Gilmour K. M. (2024). Effects
of serta and sertb knockout on aggression in zebrafish (Danio rerio). J. Comp. Physiol. A.

[43] Wang Y., Takai R., Yoshioka H., Shirabe K. (2006). Characterization and
expression of serotonin transporter genes in zebrafish. Tohoku J. Exp. Med..

[44] Vindas M. A., Gorissen M., Hoglund E., Flik G., Tronci V., Damsgard B., Thornqvist P. O., Nilsen T. O., Winberg S., Overli O., Ebbesson L. O. E. (2017). How
do individuals cope with stress?
Behavioural, physiological and neuronal differences between proactive
and reactive coping styles in fish. J. Exp.
Biol..

[45] Roubert C., Sagne C., Kapsimali M., Vernier P., Bourrat F., Giros B. A. (2001). Na­(+)/Cl­(−)-dependent
transporter for catecholamines,
identified as a norepinephrine transporter, is expressed in the brain
of the teleost fish medaka (*Oryzias latipes*). Mol. Pharmacol..

[46] Chou M. Y., Amo R., Kinoshita M., Cherng B. W., Shimazaki H., Agetsuma M., Shiraki T., Aoki T., Takahoko M., Yamazaki M., Higashijima S., Okamoto H. (2016). Social conflict resolution
regulated by two dorsal habenular subregions in zebrafish. Science.

[47] Lal P., Tanabe H., Suster M. L., Ailani D., Kotani Y., Muto A., Itoh M., Iwasaki M., Wada H., Yaksi E., Kawakami K. (2018). Identification
of a neuronal population
in the telencephalon essential for fear conditioning in zebrafish. BMC Biol..

[48] Yamamoto K., Ruuskanen J. O., Wullimann M. F., Vernier P. (2011). Differential expression
of dopaminergic cell markers in the adult zebrafish forebrain. J. Comp. Neurol..

[49] Schwarz L. A., Luo L. (2015). Organization of the
locus coeruleus-norepinephrine system. Curr.
Biol..

[50] Solis E., Zdravkovic I., Tomlinson I. D., Noskov S. Y., Rosenthal S. J., De Felice L. J. (2012). 4-(4-(dimethylamino)­phenyl)-1-methylpyridinium
(APP+) is a fluorescent substrate for the human serotonin transporter. J. Biol. Chem..

[51] Tohyama S., Miyagawa S., Lange A., Ogino Y., Mizutani T., Ihara M., Tanaka H., Tatarazako N., Kobayashi T., Tyler C. R., Iguchi T. (2016). Evolution
of estrogen
receptors in ray-finned fish and their comparative responses to estrogenic
substances. J. Steroid Biochem. Mol. Biol..

[52] Miyagawa S., Lange A., Hirakawa I., Tohyama S., Ogino Y., Mizutani T., Kagami Y., Kusano T., Ihara M., Tanaka H., Tatarazako N., Ohta Y., Katsu Y., Tyler C. R., Iguchi T. (2014). Differing
species responsiveness
of estrogenic contaminants in fish is conferred by the ligand binding
domain of the estrogen receptor. Environ. Sci.
Technol..

[53] Martin J. M., Bertram M. G., Saaristo M., Ecker T. E., Hannington S. L., Tanner J. L., Michelangeli M., O’Bryan M. K., Wong B. B. M. (2019). Impact of the widespread pharmaceutical pollutant fluoxetine
on behaviour and sperm traits in a freshwater fish. Sci. Total Environ..

[54] Weinberger J., Klaper R. (2014). Environmental
concentrations of the
selective serotonin reuptake inhibitor fluoxetine impact specific
behaviors involved in reproduction, feeding and predator avoidance
in the fish Pimephales promelas (fathead minnow). Aquat. Toxicol..

[55] Gould S. L., Winter M. J., Norton W. H. J., Tyler C. R. (2021). The potential
for
adverse effects in fish exposed to antidepressants in the aquatic
environment. Environ. Sci. Technol..

