# Supplementary material for: Characterization of Fish Serotonin, Dopamine and Norepinephrine Transporters as a Potential Target for Environmental Pharmaceuticals
Source: Environ Sci Technol. 2026 Aug 24;60(35):24580–90. doi: 10.1021/acs.est.6c04982 (PMC13564422; doi:10.1021/acs.est.6c04982)
Supplement: Supplementary file 1 [file es6c04982_si_001.pdf]

Supplement data for:

Characterization of fish serotonin, dopamine and norepinephrine transporters as a potential target for environmental pharmaceuticals

Kikuko Honda[a], Minguang Han[b,c], Fuyuka Mori[a], Ayaka Morinaga[a], Yuka Nishimura[a], Renji Kaneko[a], Kie Oizumi[a], Hana Kajiyama[a], Mariko O. Ihara[c,d], Han Zhang[c,e], Daisuke Kato[c], Kenji Toyota[a,f], Anke Lange[g], Charles R. Tyler[g], Taisen Iguchi[h], Yuji Mushirobira[d], Masaki Nagae[i], Kiyoshi Soyano[j], Masaru Ihara[c,d,#], Shinichi Miyagawa[a,k,#]

[a]Department of Biological Science and Technology, Faculty of Advanced Engineering, Tokyo University of Science, Tokyo 125-8585, Japan.
[b]Department of Environmental Engineering, Graduate School of Engineering, Kyoto University, Kyoto 615-8246, Japan
[c]Research Center for Environmental Quality Management, Kyoto University, Shiga 520-0811, Japan
[d]Faculty of Agriculture and Marine Science, Kochi University, Kochi 783-8502, Japan
[e]Key Laboratory of Industrial Ecology and Environmental Engineering (Ministry of Education), Dalian Key Laboratory on Chemicals Risk Control and Pollution Prevention Technology, School of Environmental Science and Technology, Dalian University of Technology, Dalian, Liaoning 116024, China
[f]Graduate School of Integrated Sciences for Life, Hiroshima University, Hiroshima 739-8528, Japan
[g]Biosciences, Faculty of Health and Life Sciences, University of Exeter, Exeter EX4 4QD, UK
[h]Graduate School of Nanobioscience, Yokohama City University, Kanagawa 236-0027, Japan
[i]Graduate School of Fisheries and Environmental Sciences, Nagasaki University, Nagasaki 852-8521, Japan
[j]Institute for East China Sea Research, Organization for Marine Science and Technology, Nagasaki University, Nagasaki 851-2213, Japan
[k]Division of Biological Fluctuation, Research Institute for Science and Technology (RIST), Tokyo University of Science, Tokyo 125-8585, Japan

#Corresponding author: miyagawa@rs.tus.ac.jp (Shinichi Miyagawa), ihara.masaru@kochi-u.ac.jp (Masaru Ihara)

20 pages

Page S2. Supplemental method S1.
Page S3-4. Supplemental figure legends S1-S8.
Page S5. Figure S1. The N-terminal sequences of olSERTb transcription variants.
Page S6-9. Figure S2. Sequence comparison for medaka (*O. latipes*), ayu (*P. altivelis*), zebrafish (*D. rerio*) and human (*H. sapiens*) monoamine transporters.
Page S10. Figure S3. Evolutionary relationships between chordate monoamine transporters.
Page S11. Figure S4. Distribution of olSERTa and olSERTb transcripts in adult medaka.
Page S12. Figure S5. APP uptake by medaka monoamine transporters in vitro assay.
Page S13-21. Figure S6. APP uptake inhibition by pharmaceutical antidepressants for medaka and ayu monoamine transporters.
Page S22. Figure S7. IC_{50} values of antidepressants against ayu MATs.
Page S23. Figure S8. IC_{50} values of antidepressants against hsNET, olNET, and paNET.
Page S24. Figure S9. IC_{50} values of antidepressants against hsDAT, olDAT, and paDAT.
Page S25. Table S1. Sequence of primers for cloning.
Page S26-27. Table S2. Accession numbers of the sequences used in the phylogenetic analysis.
Page S28. Table S3. List of test chemicals used in this study.

Supplemental methods

Methods S1

***In situ* hybridization for gene expression analysis**

Twenty µm sections were deparaffinized, rehydrated, incubated in 1 µg/ml proteinase K for 7 min at 37˚C and refixed with 4% paraformaldehyde (PFA) for 10 min at room temperature. After washing in PBS containing 0.1% Tween-20, an overnight hybridization was performed in a formamide buffer (50% formamide, 5x saline sodium citrate, 50 µg/ml yeast tRNA, 1% sodium dodecyl sulfate, 50 µg/ml heparin) with 1 µg/ml probe at 68˚C. After washing with 5x saline sodium citrate and 50% formamide for 1 h at 68˚C, the slides were incubated in blocking solution [10% blocking reagent (Roche, Basel, Switzerland) in 100 mM maleate buffer and TBST (140 mM NaCl, 2.7 mM KCl, 0.1% Tween-20, 25 mM Tris-HCl, pH 7.5)] for 2 h. Anti-digoxigenin antibody (Roche) in a blocking solution was then added to the slides and incubated for 1 hr. After washing with TBST, the sections were equilibrated in NTMT buffer (100 mM NaCl, 50 mM $MgCl_2$, 0.1% Tween-20 and 100 mM Tris-HCl, pH 9.5) including 2 mM levamisole (Sigma-Aldrich) and incubated in color solution [3.5 µg nitrotetrazolium blue chloride (NBT; Sigma-Aldrich) and 1.75 µg 5-Bromo-4-chloro-3-indolyl phosphate (BCIP; Sigma-Aldrich) per milliliter of NTMT buffer].

Supplementary figure legends

Fig. S1

The N-terminal sequences of olSERTb transcription variants. Two *Oryzias latipes* SERTb (olSERTb) splice variants (olSERTbx1 and olSERTbx2) differ in their N-terminal sequence.

Fig. S2

Sequence comparison for medaka (*O. latipes*), ayu *(P. altivelis*), zebrafish (*D. rerio*) and human (*H. sapiens*) monoamine transporters. Multiple sequence alignments of the SERT (A), NET (B), and DAT (C) proteins were carried out using MUSCLE program. The amino acid residues that are predicted to form the binding sites for sodium and chloride ions are indicated by *. The amino acid residues that are predicted to interact with endogenous substrates (i.e., 5-HT for SERT, norepinephrine for NET, and dopamine for DAT, respectively) are in red. Among them, the amino acids that are not conserved are marked with boxes. Prefixes in gene names indicate the species of origin: ol, *Oryzias latipes* (medaka); pa, *Plecoglossus altivelis* (ayu); dr, *Danio rerio* (zebrafish); hs, *Homo sapiens* (human).

Fig. S3

Evolutionary relationships between chordate monoamine transporters. A maximum likelihood tree was constructed using a 1000 replicate bootstrap analysis and the tree with the highest log likelihood is shown. The percentage of trees in which the associated taxa clustered together is shown next to the branches. The branch length reflects the estimated proportions of substitutions along each branch. The scale bar represents 0.2 substitutions per site. Since protostome DAT (invertebrate DAT) is not an ortholog of chordate DAT [17142674], we use drosophila DAT (NP_523763), was used as a outgroup of the phylogeny. The lamprey DAT-like sequence [XP_032834816] was not used in this phylogenetic analysis because only partial sequences were registered.

Fig. S4

Distribution of olSERTa and olSERTb transcripts in adult medaka. RT-PCR was performed using total RNA isolated from various tissues of adult medaka. Elongation factor 1a (EF1a) was amplified as an internal control.

Fig. S5

APP uptake by medaka monoamine transporters *in vitro* assay. The efficiency of APP uptake is measured as APP fluorescence inside cells (AFU=arbitrary fluorescence unit) for O*ryzias latipes* SERTa (olSERTa), olSERTb, olSERTb transcription variant x2, olNET, and olDAT. The empty pcDNA3.1 vector is the negative control. Error bars indicate SEM.

Fig. S6

APP uptake inhibition by pharmaceutical antidepressants for medaka and ayu monoamine transporters. Effect of antidepressants on the inhibition of APP uptake is evaluated as APP fluorescence inside cells (APP uptake relative to maximum, %) for the O*ryzias latipes* SERTa (olSERTa), olNET, olDAT, *Plecoglossus altivelis* SERTa (paSERTa), paNET and paDAT. Error bars indicate SEM.

Fig. S7

Comparative IC50 values of antidepressants against ayu MATs. Plots indicate IC50 values (M) of antidepressants measured in this study. Each transporter is represented by a different symbol: *Plecoglossus altivelis* SERTa (paSERTa), black square; paNET, red triangle; and paDAT, blue circle.

Fig. S8

Comparative IC50 values of antidepressants against hsNET, olNET, and paNET. Plots indicate IC50 values (M) of antidepressants measured in this study. Each transporter is represented by a different symbol: hsNET, black square, olNET, blue circle; and paSERTa, orange triangle. Prefixes denote species: hs, *Homo sapiens*; ol, *Oryzias latipes*; pa, *Plecoglossus altivelis*.

Fig. S9
Comparative IC50 values of antidepressants against hsDAT, olDAT, and paDAT. Plots indicate IC50 values (M) of antidepressants measured in this study. Each transporter is represented by a different symbol: hsDAT black square, olDAT, blue circle; and paDAT, orange triangle. Prefixes denote species: hs, *Homo sapiens*; ol, *Oryzias latipes*; pa, *Plecoglossus altivelis*.

Fig. S1

```
olSERTbx1 --------MPRQDSAGMAGSLAHQGSPNPGYNAHNPVTVPVPTQTDSRDKWSKKMDFLLS
olSERTbx2 MELPFLHLSPLERRPLLFTPLLSRGDP-----LEDPDLCSSPCSS---------------

olSERTbx1 VIGFAVDLGNVWRFPYICYQNGGGAFLIPYILMAIFGGVPLFYMELALGQFHRTGAISIW
olSERTbx2 -----------WETPLSGMSGSEGAFLIPYILMAIFGGVPLFYMELALGQFHRTGAISIW

olSERTbx1 KHICPIFKGIGYAICIIALYVSFYYNTIIAWALFYFYSSFSTILPWTNCDNAWNTPDCTN…
olSERTbx2 KHICPIFKGIGYAICIIALYVSFYYNTIIAWALFYFYSSFSTILPWTNCDNAWNTPDCTN…
```

Fig. S2A

```
hsSERT             1 METTP--------------LNSQKQLSACEDGEDCQENGVLQKV---VPTPGDK-VESG-    41
olSERTa            1 MESKDTMMTSMQTKDKQGYEEDKGSKQPEAQEEEPHENANLPLVDG-LADRGPKSVTSGS    59
paSERTa            1 MEMKPPSTTTMMMMER---ENEGEREKAEEENEGTRENGRLMLADGSLGDKDPK-LTSGS    56
drSERTa            1 MDMKESM------------MMNQEYGGEQQKVPESQENGRLVVDS--VPEKDQK-SGSGP    45
olSERTbx1          1 --------------------------MPRQDSAGMAGS---------LAHQGSP------    19
olSERTbx2          1 ------------------MELPFLHLSPLERRPLLFTP---------LLSRGDP------    27
paSERTb            1 --------------------------MPRQDSTAPVGN---------SCAQVSS------    19
drSERTb            1 --------------------------MPHQEQVIAHGN---------LCAPGPP------    19

hsSERT            42 --QISNGYSAVPSP------------------GAGDDTRHSIP-ATTTTLVAELHQGE--    78
olSERTa           60 GQQVSNGFTTST-PQSPREALGTAVGAVGMPPASGGDTGASAPFGGLRTLVVQQTSLDRP   118
paSERTa           57 G-QVSNGFPSSTSPQSPRE-------------GAGGSTGGQGP---LRTLVVQQTSLERP    99
drSERTa           46 G-QVSNGYRSTS-PQSPKE-------------GAGTGTDRVNTPGTFRTLVVQQTSLDPP    90
olSERTbx1         20 ----NPGYNAHN-P-----------------------------------VTVPVPTQTDS    39
olSERTbx2         28 --LEDPDLCSSPCS----------------------------------------------    39
paSERTb           20 ----NASYNTNNAP-VP--------------------------------VITQTDS----    38
drSERTb           20 ---NNAGYNSNPVP-----------------------------------VIIQTES----    37

                                    * ***  *                   *
hsSERT            79 RETWGKKVDFLLSVIGYAVDLGNVWRFPYICYQNGGGAFLLPYTIMAIFGGIPLFYMELA   138
olSERTa          119 RETWSKKMDFLLSVIGYAVDLGNVWRFPYICYQNGGGAFLLPYLLMAVFGGVPLFYMELA   178
paSERTa          100 RETWSKKMDFLLSVIGYAVDLGNVWRFPYICYQNGGGAFLLPYLVMAVFGGVPLFYMELA   159
drSERTa           91 RETWSKKMDFLLSVIGYAVDLGNVWRFPYICYQNGGGAFLLPYLLMAVFGGVPLFYMELA   150
olSERTbx1         40 RDKWSKKMDFLLSVIGFAVDLGNVWRFPYICYQNGGGAFLIPYILMAIFGGVPLFYMELA    99
olSERTbx2         40 -----------------------SWETPLSGMSGSEGAFLIPYILMAIFGGVPLFYMELA    76
paSERTb           39 RDKWSKKMDFLLSVIGFAVDLGNVWRFPYICYQNGGGAFLIPYILMAIFGGVPLFYMELA    98
drSERTb           38 RDKWSKKMDFLLSVIGFAVDLGNVWRFPYICYQNGGGAFLIPYVLMAVFGGVPLFYMELA    97

hsSERT           139 LGQYHRNGCISIWRKICPIFKGIGYAICIIAFYIASYYNTIMAWALYYLISSFTDQLPWT   198
olSERTa          179 LGQFHRSGCISIWKHICPIFKGIGFAICIIALYIAFYYNTIMAWALYYLLSSFRPTLPWT   238
paSERTa          160 LGQFHRSGCISIWKHICPIFKGIGFAICIIALYIAFYYNTIMAWALYYLLSSFRPTLPWT   219
drSERTa          151 LGQFHRSGCISIWKHVCPIFKGIGFAICIIALYIAFYYNTIMAWALYYLLSSFRATLPWT   210
olSERTbx1        100 LGQFHRTGAISIWKHICPIFKGIGYAICIIALYVSFYYNTIIAWALFYFYSSFSTILPWT   159
olSERTbx2         77 LGQFHRTGAISIWKHICPIFKGIGYAICIIALYVSFYYNTIIAWALFYFYSSFSTILPWT   136
paSERTb           99 LGQFHRTGAISIWKHICPIFKGIGFAICIIALYVSFYYNTIIAWALFYFYSSFASTLPWT   158
drSERTb           98 LGQFHRTGAISIWKHICPIFKGIGFAICIIALYVSFYYNTIIAWALFYFYSSFSSTLPWT   157

hsSERT           199 SCKNSWNTGNCTNYFSED-NITWTLHSTSPAEEFYTRHVLQIHRSKGLQDLGGISWQLAL   257
olSERTa          239 TCTNSWNTVNCYRYLSSDQNVTWSNLSTSPAEEFYTRQVLQVHLSSGLHQLGSVSWQLAL   298
paSERTa          220 TCSNSWNTVNCLRYLSTDQNVTWTNSSTSPAEEFYTRQVLQVHLSPGLHQLGAVSWQLAL   279
drSERTa          211 TCNNRWNTPNCTHYLSTDLNVSWTNNSISPAEEFYVRQVLQVHLSPGLHQLGWVSWQLAL   270
olSERTbx1        160 NCDNAWNTPDCTNYFGMD-NVTWTNSSRSPAEEFYTRNVLEIHKSSGLKNVGGVRWQLLL   218
olSERTbx2        137 NCDNAWNTPDCTNYFGMD-NVTWTNSSRSPAEEFYTRNVLEIHKSSGLKNVGGVRWQLLL   195
paSERTb          159 NCDNVWNTENCTNYFGKD-NVTWNNYSRSPAEEFYTRNVLEIHKSPGLHNVGTVRWQLML   217
drSERTb          158 SCDNDWNTENCTNYFGKD-NVTWTNYSRSPAEEFYTRNVLAVHESSGLGNVGYIRWQLML   216

hsSERT           258 CIMLIFTVIYFSIWKGVKTSGKVVWVTATFPYIILSVLLVRGATLPGAWRGVLFYLKPNW   317
olSERTa          299 CLLFIFTIVYFSIWKGVKTSGKVVWVTATFPYLVLLILLIRGATLPGAWRGVVFYLKPDW   358
paSERTa          280 CLLFIFTIVYFSIWKGVKTSGKVVWVTATFPYLVLLILLVRGATLPGAWRGVVFYLKPDW   339
drSERTa          271 CLLFIFTVVYFSIWKGVKTSGKVVWVTATFPYLVLLILLIRGATLPGAWRGVVFYLKPDW   330
olSERTbx1        219 CLFLIFTIVYFSLWKGVKTSGKVVWVTATLPYIVLFILLIRGATLPGAWRGVVFYLKPQW   278
olSERTbx2        196 CLFLIFTIVYFSLWKGVKTSGKVVWVTATLPYIVLFILLIRGATLPGAWRGVVFYLKPQW   255
paSERTb          218 CLFLIFTIVYFSLWKGVKTSGKVVWVTATLPYVVLFILLIRGATLPGAWRGVVFYLKPKW   277
drSERTb          217 CLFLIFTIVYFSLWKGVKTSGKVVWVTATLPYVVLLILMIRGATLPGAWKGVVFYLNPKW   276
```

Fig. S2A (continue)

```
                                   *                               *   *
hsSERT       318 QKLLETGVWIDAAAQIFFSLGPGFGVLLAFASYNKFNNNCYQDALVTSVVNCMTSFVSGF 377
olSERTa      359 QKLLSTTVWIDAAAQIFFSLGPGFGVLLAFASYNPFHNNCYKDALVTSSVNCLTSFLSGF 418
paSERTa      340 EKLLSTTVWIDAAAQIFFSLGPGFGVLLAFASYNPFHNNCYKDALVTSSVNCLTSFLSGF 399
drSERTa      331 KKLLTTTVWLDAAAQIFFSLGPGFGVLLAFASYNPFHNNCYKDALITSSVNCLTSFLSGF 390
olSERTbx1    279 EKLLETSVWVDAAAQIFFSLGPGFGVLLALSSYNPFTNNCYRDAIVTSLVNCLTSFVSGF 338
olSERTbx2    256 EKLLETSVWVDAAAQIFFSLGPGFGVLLALSSYNPFTNNCYRDAIVTSLVNCLTSFVSGF 315
paSERTb      278 EKLLETSVWVDAAAQIFFSLGPGFGVLLALSSYNPFTNNCYRDAIVTSLVNCLTSFISGF 337
drSERTb      277 EKLKETSVWVDAAAQIFFSLGPGFGVLLALSSYNPFTNNCYRDAIVTSLVNCLTSFVSGF 336

                                                                         *  *
hsSERT       378 VIFTVLGYMAEMRNEDVSEVAKDAGPSLLFITYAEAIANMPASTFFAIIFFLMLITLGLD 437
olSERTa      419 VIFTVLGYMAEMRQQNVDVVAKDAGPSLLFIIYAEAIANMPAATFFAIIFFLMIIMLGLD 478
paSERTa      400 VIFTVLGYMAEMRQQEVESVAKDAGPSLLFIIYAEAISNMPAATFFAIIFFLMIIMLGLD 459
drSERTa      391 VIFTVLGYMAEMRQQGVETVAKDAGPSLLFIIYAEAIANMPAATFFAIIFFLMIIMLGLD 450
olSERTbx1    339 VIFTVLGYMAEMRKVEVEDVAKDKGPSLLFITYPEAIANMMGSTFFAIIFFVMMISLGLD 398
olSERTbx2    316 VIFTVLGYMAEMRKVEVEDVAKDKGPSLLFITYPEAIANMMGSTFFAIIFFVMMISLGLD 375
paSERTb      338 VIFTVLGYMAEMRKVEVEDVARDKGPSLLFITYPEAIANMMGSTFFAIIFFVMMITLGLD 397
drSERTb      337 VIFTVLGYMAEQRNVNVEDVARDKGPSLLFITYPEAIANMVGSTFFAIIFFVMMITLGLD 396

                 *
hsSERT       438 STFAGLEGVITAVLDEFPHVWAKRRERFVLAVVITCFFGSLVTLTFGGAYVVKLLEEYAT 497
olSERTa      479 STFAGLEGVITAMLDEFPRLLAKRREWFVFGLVCVCYLGALSTLTYGGAFVVKLFEEYAT 538
paSERTa      460 STFAGLEGVITAMLDEFPHLLAKRRECFVLGLVCVCYLGALSTLTYGGAFVVKLFEEYAT 519
drSERTa      451 STFAGLEGVITAMLDEFPHLLARRREWFVLGLVCVCYLGALSTLTYGGAFVVKLFEEYAT 510
olSERTbx1    399 STFGGLEAIITAVLDEYPDQLSHRRELFVLCLVVVCFLGSLSTLTYGGAYVVKLLEEFGV 458
olSERTbx2    376 STFGGLEAIITAVLDEYPDQLSHRRELFVLCLVVVCFLGSLSTLTYGGAYVVKLLEEFGV 435
paSERTb      398 STFGGLEAIITAVLDEYPGSLSHRRELFVLGLVIVCFLGSLSTLTNGGAYVVKLLEEFGV 457
drSERTb      397 STFGGLEAIITAVMDEYPDVLSHRRELFVLGLVVVCFLGSLSTLTKGGAYVVKLLEEFGV 456

hsSERT       498 GPAVLTVALIEAVAVSWFYGITQFCRDVKEMLGFSPGWFWRICWVAISPLFLLFIICSFL 557
olSERTa      539 GPAVITVVLLEVIAVSWFYGTSRFCNDIQVMLGFYPGCFWRVCWVAICPCFLLFIIISFL 598
paSERTa      520 GPAVITVVFLEVIAVSWFYGTTRFCSDVKLMLGFSPGIFWRVCWVAICPCFLLFIIVSFL 579
drSERTa      511 GPAVITVVFLEVIAVSWFYGTTRFCNDVQLMLGFAPGLFWRVCWIAICPCFLLFIIVSFL 570
olSERTbx1    459 GCSIIAVGFLEAIAVSWFYGINRFSNDVQAMLGKAPGLFWRVCWVGISPAFLAYIIVSSL 518
olSERTbx2    436 GCSIIAVGFLEAIAVSWFYGINRFSNDVQAMLGKAPGLFWRVCWVGISPAFLAYIIVSSL 495
paSERTb      458 GSSIIAVGFLEVIAVSWFYGIQRFSNDIQAMLGYAPGLFWKVCWVAISPAFLAYIIVSSL 517
drSERTb      457 GSSIIAIVFLEATAVSWFYGINRFSNDIKSMLGYTPGLFWKVCWVAISPAFLAYIIISSL 516

hsSERT       558 MSPPQLRLFQYNYPYWSIILGYCIGTSSFICIPTYIAYRLIITPGTFKERIIKSITPETP 617
olSERTa      599 AFPPEVKLFNYTYPPWTTVLGYCIGVSSFICVPSYMVYYLLNAKGTFKQRLLKSITPE-- 656
paSERTa      580 IFPPEVTLFDYHYPAWTTVLGYCIGLSSFICVPSYMVFHLLTSKGTLKQRLLKGITPEA- 638
drSERTa      571 AFPPEVKLFDYLYPFWTTVLGYCIGVSSFICVPSYMVYHLVTTKGTFQQRLLKGITPEAP 630
olSERTbx1    519 LKAPPLTLFDYKYPDWSITVGYIIGFSSFMWIPIYMVYKLVWTPGSLKQRLAVCLRPER- 577
olSERTbx2    496 LKAPPLTLFDYKYPDWSITVGYIIGFSSFMWIPIYMVYKLVWTPGSLKQRLAVCLRPER- 554
paSERTb      518 LKPPPLTLFDYKYPDWSISVGYVIGASSFIWIPVYMVYKLVWTPGSLKQRLAVCLRPER- 576
drSERTb      517 LNPQTLTLFDYEFPDWSITVGYIIGASSFIWIPIYMVYKLVWTPGSLKQRLAVCLRPER- 575

hsSERT       618 TEIPCGDIR-----LNAV-                                          630
olSERTa      657 ---PSSENHRDYIVTNAI-                                          671
paSERTa      639 ---SCGSQR-DGIVTNAV-                                          652
drSERTa      631 G--SSGPQRDTIVITNAV-                                          646
olSERTbx1    578 ---TIPDIHADNLNMTIVQ                                          593
olSERTbx2    555 ---TIPDIHADNLNMTIVQ                                          570
paSERTb      577 ---TVPDIHTDNLNMTPIP                                          592
drSERTb      576 ---TLPDIHTDGMSLSPVP                                          591
```

Fig. S2B

```
                                    *  ***  *                   *
hsNET   46 LAPRDGDAQPRETWGKKIDFLLSVVGFAVDLANVWRFPYLCYKNGGGAFLIPYTLFLIIA  105
```

```
hsNET    1 MLLARMNPQVQPENNGADTGPEQPLRA-RKTAELLVVKERNGVQ--------------CL   45
olNET    1 -----MNIQVLPEHKLSSVAPLKQCNVEKKEVELILVKDQNGVQYTNSTII--PTAPRCA   53
paNET    1 -----MNIQVLPEHKLSSVAPLKQCNVEKKEVELILVKDQNGVQYTSSSVI--PN---CS   50
drNET    1 -----MNIQVLPEHKLSSVAPLKQFNVEKKEVELILVKEQNGVQYTNSSVVASPSSRACT   55

                                    *  ***  *                   *
hsNET   46 LAPRDGDAQPRETWGKKIDFLLSVVGFAVDLANVWRFPYLCYKNGGGAFLIPYTLFLIIA  105
olNET   54 GPHGTSTDIERETWGKKIDFLLSVIGFAVDLANVWRFPYLCYKNGGGAFLIPYILFLVIA  113
paNET   51 G--GPPEDDERETWGKKIDFLLSVIGFAVDLANVWRFPYLCYKNGGGAFLIPYILFLVIA  108
drNET   56 S--ASGEEIERETWGKKMDFLLSVIGFAVDLANVWRFPYLCYKNGGGAFLIPYVLFLFIA  113

hsNET  106 GMPLFYMELALGQYNREGAATVWKICPFFKGVGYAVILIALYVGFYYNVIIAWSLYYLFS  165
olNET  114 GMPLFYMELALGQYNREGAATVWKICPVFKGVGYAVIVIALYVGFYYNVIIAWSLYYLYS  173
paNET  109 GMPLFYMELALGQYNREGAATVWKICPVFKGVGYTVIIIALYVGFYYNVIIAWSLYYLFS  168
drNET  114 GMPLFYMELALGQYNREGAATLWKICPVFKGVGYTVIVIALYVGFYYNVIIAWSLYYLYA  173

hsNET  166 SFTLNLPWTDCGHTWNSPNCTDPKLLNGSVLGNHTKYSKYKFTPAAEFYERGVLHLHESS  225
olNET  174 SMTAELPWLKCGHSWNSINCTDPQHINASFFDKNTSYANNKFTPASEYYERGVLHLHESK  233
paNET  169 SMTGELPWLHCRNSWNSENCTDPKLFNGSFLANGTSYAKYKITPAAEYYERGVLHLHESR  228
drNET  174 SLTSELPWLHCNNPWNSPNCTEPKDINGTVLGNGTSYAKYKNTPAAEFYERGVLHSHESK  233

hsNET  226 GIHDIGLPQWQLLLCLMVVVIVLYFSLWKGVKTSGKVVWITATLPYFVLFVLLVHGVTLP  285
olNET  234 GIQDLGLPRLELTLCLIVVVFILYFSLWKGVKSSGKVVYITATMPYVVLFVLLIRGITLP  293
paNET  229 GIHDLGLPRWELTLCLVVVVFILYFSLWKGVKSSGKVVYITATMPYVVLFVLLIRGITLP  288
drNET  234 GIGDLGLPRWELTICLVVVAFILYFSLWKGVKSSGKVVYITATMPYIVLLVLLIRGITLP  293

                                           *
hsNET  286 GASNGINAYLHIDFYRLKEATVWIDAATQIFFSLGAGFGVLIAFASYNKFDNNCYRDALL  345
olNET  294 GALNGIKAYLNFSLSELNNLQVWIDAATQIFYSLGAGFGVLIAFSSYNKFDNNCYRDAIL  353
paNET  289 GAMDGIRAYLHIDFNMLNNPKVWIDAATQIFYSLGAGFGVLIAFASYNKFDNNCYRDALL  348
drNET  294 GAMNGIRAYLHIDLKKLNNPQVWIDAATQIFFSLGAGFGVLIAFSSYNKFNNNCYRDAIL  353

               *   *
hsNET  346 TSSINCITSFVSGFAIFSILGYMAHEHKVNIEDVATEGAGLVFILYPEAISTLSGSTFWA  405
olNET  354 TSAVNCITSFVSGFAIFSVLGYMAQKYNMDIKAVATDGPGLVFIIYPEAISTLPGSTFWA  413
paNET  349 TSTVNCVTSFFSGFAIFSVLGYMADKHGVKIEDVATEGAGLVFIIYPEAISTLPGSTFWA  408
drNET  354 TSTINCVTSFFSGFAIFSVLGYMAEQHKVNIKDVATEGAGLVFVIYPEAISTLPGATFFA  413

                    *  **
hsNET  406 VVFFVMLLALGLDSSMGGMEAVITGLADDFQVLKRHRKLFTFGVTFSTFLLALFCITKGG  465
olNET  414 IVFFIMLLTLGIDSSMGGMEAVITGLSDDFKFLKRNRKLFTFATAFGTFLMALICVTNGG  473
paNET  409 IVFFIMLLTLGIDSSMGGMEAVITGLTDDVKILKRNRKLFTFATAFGTFLVALFCITNGG  468
drNET  414 IVFFIMLLTLGIDSSMGGMEAVLTGLSDDFKILKRNRKLFTFATVVGTFLMALLCVTNGG  473

hsNET  466 IYVLTLLDTFAAGTSILFAVLMEAIGVSWFYGVDRFSNDIQQMMGFRPGLYWRLCWKFVS  525
olNET  474 IYVLTLLDKYAAGTSILFAVLIEAIGVSWFYGVDRFSEDIEQMMGFKPGLYWRLCWKFVS  533
paNET  469 IYVLTLLDNYAAGTSILFAVLIEAIGVSWFYGVDRFSEDIERMMGFKPGLYWRLCWKFVS  528
drNET  474 IYVLTLLDKYAAGTSILFGVLFEAIGVSWFYGVDRFSEDIERMMGFKPGIYWRLCWKFVS  533

hsNET  526 PAFLLFVVVVSIINFKPLTYDDYIFPPWANWVGWGIALSSMVLVPIYVIYKFLSTQGSLW  585
olNET  534 PVFLLFVVIASTVTSSGLKYGDYVFPNGANLLGWGVAMSSMLFVPFYAIYKFFSVRGTFT  593
paNET  529 PAFLLFVVVASIVTSTGLSYDDYVFPQWSNVVGWGVAMSSMLFVPIYAIYKFCSVSGTFK  588
drNET  534 PAFLLLVVIASIVTSGRLTYDEYVFPYWADMLGWSIALSSMLFVPFYAIYKFFSVPSSFK  593

hsNET  586 ERLAYGITPENEHHLVAQRDIRQFQLQHWLAI                              617
olNET  594 ERIAYCITPECEHHKVAEGNVRQFKLKHWLAI                              625
paNET  589 QRIAYCITPEHEHHLVAEGNIRQFTLKHWLAI                              620
drNET  594 ERLAYCVTPEHEHHLVAEGNVRQFKLKHWLTI                              625
```

Fig. S2C

```
hsDAT    1 --MSKSKCSVGL----MSSVVAPAK-EPNAVGPKEVELILVKEQNGVQLTSSTLTNPR--    51
olDAT    1 ----------------MSSVIVPEKPSSNTMGPKEVELILVKEQNGVQFTSTTLVTPAAP    44
paDAT    1 --MLRARVPSGQ----MCSVVAPAQPGTNTMGPRDVELILVKEQNGVQFTSSTLVNPA--    52
drDAT    1 MPMLRGRPAVTHTRTRTHTHMSSVSGSSSAAGPREVELVLVKEQNGVQFTSSSLRNPGAH    60

                                         * ***  *                   *
hsDAT   52 -------QSPVEAQDRETWGKKIDFLLSVIGFAVDLANVWRFPYLCYKNGGGAFLVPYLL   104
olDAT   45 Q------TSRSGEEERETWGKKIDFLLSVIGFAVDLANVWRFPYLCYKNGGGAFLVPYLF    98
paDAT   53 -------QTHPPEEERETWGKKIDFLLSVIGFAVDLANVWRFPYLCYKNGGGAFLVPYLF   105
drDAT   61 SHTHTHTHTHPSGQQRETWGKKIDFLLSVIGFAVDLANVWRFPYLCYKNGGGAFLVPYLL   120

hsDAT  105 FMVIAGMPLFYMELALGQFNREGAAGVWKICPILKGVGFTVILISLYVGFFYNVIIAWAL   164
olDAT   99 FMLIAGMPLFYMELALGQYNREGAAGVWKICPIFKGVGFTVILISLYVGFYYNVIISWAL   158
paDAT  106 FMVIAGMPLFYMELALGQYNREGAAGVWKICPIFKGVGFTVILISLYVGFYYNVIIAWAL   165
drDAT  121 FMVIAGMPLFYMELALGQYNREGAAGVWKICPIFKGVGFTVILISLYVGSYYNVIIAWAL   180

hsDAT  165 HYLFSSFTTELPWIHCNNSWNSPNCSDAHPGDSSGDSSGLNDTFGTTPAAEYFERGVLHL   224
olDAT  159 FYLFSSFTWELPWVHCNNTWNSPNCSD-WA-----DNNTVSDIYTATPAQEYFERGVLHI   212
paDAT  166 FYLFSSFTGDLPWVHCNNTWNSPNCSDVWS-----FNTTLNDTQKSTPAEEYFERGVLHL   220
drDAT  181 FYLFSSFSGELPWIHCNNTWNSPNCSD--P-----NATLLNDTYKTTPALEYFERGVLHV   233

hsDAT  225 HQSHGIDDLGPPRWQLTACLVLVIVLLYFSLWKGVKTSGKVVWITATMPYVVLTALLLRG   284
olDAT  213 QDSNGIDDLGRPRWQLTACLAVVIVLLYFCLWKGVKTSGKVVWITATMPYLVLTVLLLRG   272
paDAT  221 QDSEGIDDLGLPRWQLTSCLAVVIVVLYFSLWKGVKTSGKVVWITATMPYLVLTVLLLRG   280
drDAT  234 HESSGIDDLGAPRWQLTACLAVVIVVLYFSLWKGVKTSGKVVWITATMPYVVLTVLLLRG   293

                                                *
hsDAT  285 VTLPGAIDGIRAYLSVDFYRLCEASVWIDAATQVCFSLGVGFGVLIAFSSYNKFTNNCYR   344
olDAT  273 VTLPGAIDGIKAYLSVDFLRLCDAKVWIEAATQICFSLGVGFGVLIAFSSYNKFSNNCYR   332
paDAT  281 VTLPGAMDGIKAYLSVDLIKLCEAKVWIEAATQICFSLGVGFGVLIAFSSYNKFSNNCYR   340
drDAT  294 VTLPGAIDGIKAYLSVDFLRLYDAQVWIEAATQICFSLGVGFGVLIAFSSYNKFSNNCYR   353

                   *   *
hsDAT  345 DAIVTTSINSLTSFSSGFVVFSFLGYMAQKHSVPIGDVAKDGPGLIFIIYPEAIATLPLS   404
olDAT  333 DAIITSSINSLTSFFSGFVVFSFLGYMSHKHNVALDKVARDGAGLVFVIYPEAIATLPGS   392
paDAT  341 DAIITTSINSLTSFFSGFVIFSFLGYMAQKHNVALDKVAQDGPGLVFIIYPEAIATLPGS   400
drDAT  354 DAIITSSINSLTSFFSGFVIFSFLGYMSQKHNVALDKVATDGPGLVFIIYPEAIATLPGS   413

                        *   **
hsDAT  405 SAWAVVFFIMLLTLGIDSAMGGMESVITGLIDEFQLLHRHRELFTLFIVLATFLLSLFCV   464
olDAT  393 SVWAVIFFIMLLTLGIDSAMGGMESVITGLIDEFKFLHKHRELFTLFIVVSTFLISLFCV   452
paDAT  401 SVWAVIFFIMLLTLGIDSAMGGMESVITGLIDEFKCLHKHRELFTLFIVVSTFLISLFCV   460
drDAT  414 SVWAVIFFIMLLTLGIDSAMGGMESVITGLIDEFKFLHKHRELFTLFIVVSTFLISLICV   473

hsDAT  465 TNGGIYVFTLLDHFAAGTSILFGVLIEAIGVAWFYGVGQFSDDIQQMTGQRPSLYWRLCW   524
olDAT  453 TSGGMYVFTLLDHFAAGTSILFGVLIEAIGIAWFYGVDRFSDDIKEMIGHRPGRYWRLCW   512
paDAT  461 TNGGIYVFTLLDHFAAGTSILFGVLIEAIGIAWFYGVDRFSDDIEEMIGQRPGLYWRLCW   520
drDAT  474 TNGGIYVFTLLDHFAAGTSILFGVLIEAIGIAWFYGVDRFSDDIEEMIGQRPGLYWRLCW   533

hsDAT  525 KLVSPCFLLFVVVVSIVTFRPPHYGAYIFPDWANALGWVIATSSMAMVPIYAAYKFCSLP   584
olDAT  513 KFVSPCFLLFMVVVSFATFNPPNYGSYMFPPWANMLGWCLAMSSMTMVPLYAIYKLCTLP   572
paDAT  521 KFVSPCFLLFMVVVSFATFNPPTYGPYTFPVWANMIGWCLAISSMSMVPLYAIYKLCTIP   580
drDAT  534 KFVSPCFLLFMVVVSFATFNPPKYGSYYFPTWATMVGWCLSISSMIMVPLYAFYKFCSLP   593

hsDAT  585 GSFREKLAYAIAPEKDRELVDRGEVRQFTLRHWLKV                           620
olDAT  573 GKFCHRLAYAITPETEHHLVDNGEVRQFTLRHWLVV                           608
paDAT  581 GKFCDRLAYAITPETEHHLVDDGEVRQFSLHHWLVI                           616
drDAT  594 GSFCDKLAYAITPETDHHLVERGEVRQFTLHHWLVV                           629
```

Fig. S3

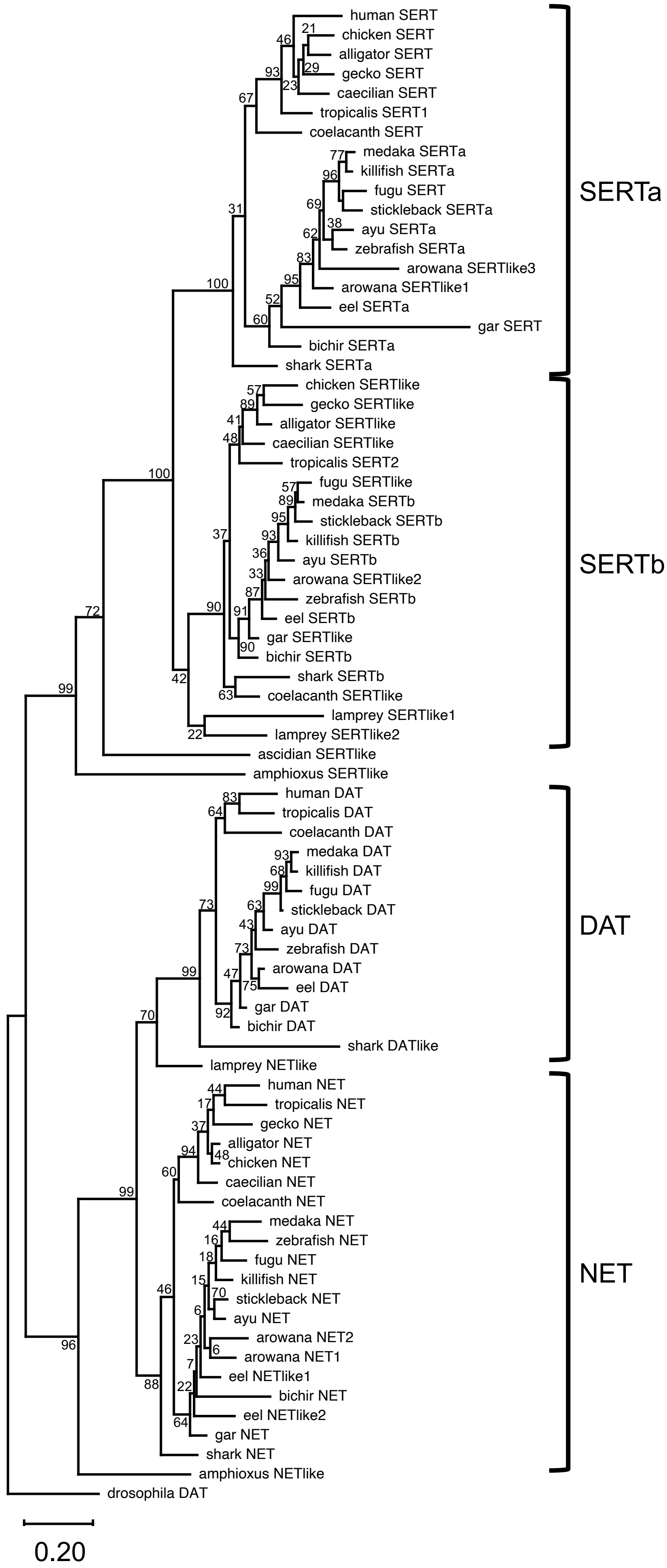
human SERT
chicken SERT
alligator SERT
gecko SERT
caecilian SERT
tropicalis SERT1
coelacanth SERT
medaka SERTa
killifish SERTa
fugu SERT
stickleback SERTa
ayu SERTa
zebrafish SERTa
arowana SERTlike3
arowana SERTlike1
eel SERTa
gar SERT
bichir SERTa
shark SERTa
chicken SERTlike
gecko SERTlike
alligator SERTlike
caecilian SERTlike
tropicalis SERT2
fugu SERTlike
medaka SERTb
stickleback SERTb
killifish SERTb
ayu SERTb
arowana SERTlike2
zebrafish SERTb
eel SERTb
gar SERTlike
bichir SERTb
shark SERTb
coelacanth SERTlike
lamprey SERTlike1
lamprey SERTlike2
ascidian SERTlike
amphioxus SERTlike
human DAT
tropicalis DAT
coelacanth DAT
medaka DAT
killifish DAT
fugu DAT
stickleback DAT
ayu DAT
zebrafish DAT
arowana DAT
eel DAT
gar DAT
bichir DAT
shark DATlike
lamprey NETlike
human NET
tropicalis NET
gecko NET
alligator NET
chicken NET
caecilian NET
coelacanth NET
medaka NET
zebrafish NET
fugu NET
killifish NET
stickleback NET
ayu NET
arowana NET2
arowana NET1
eel NETlike1
bichir NET
eel NETlike2
gar NET
shark NET
amphioxus NETlike
drosophila DAT
SERTa
SERTb
DAT
NET
0.20

Fig. S4

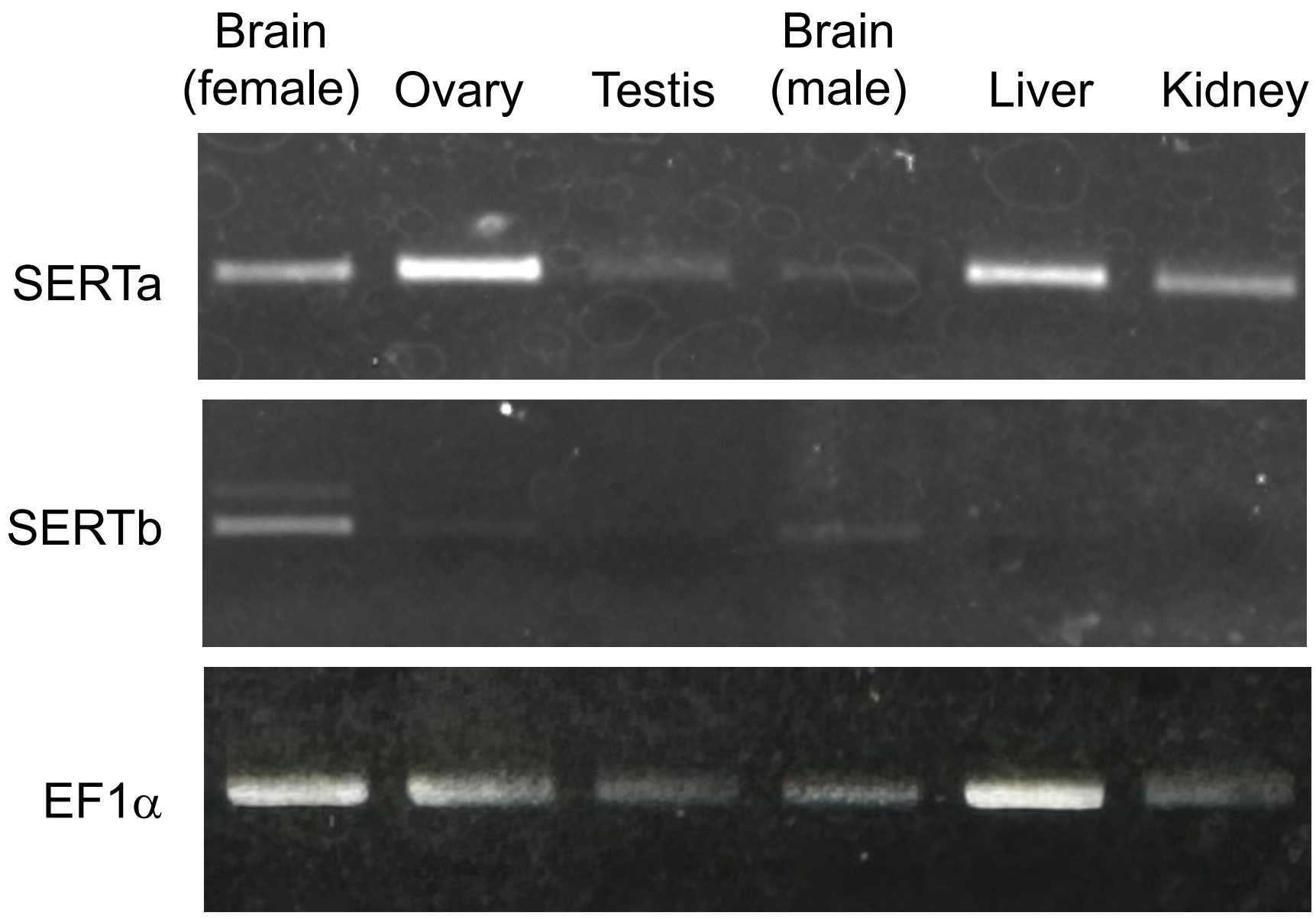

Brain (female)
Ovary
Testis
Brain (male)
Liver
Kidney
SERTa
SERTb
EF1α

Fig. S5

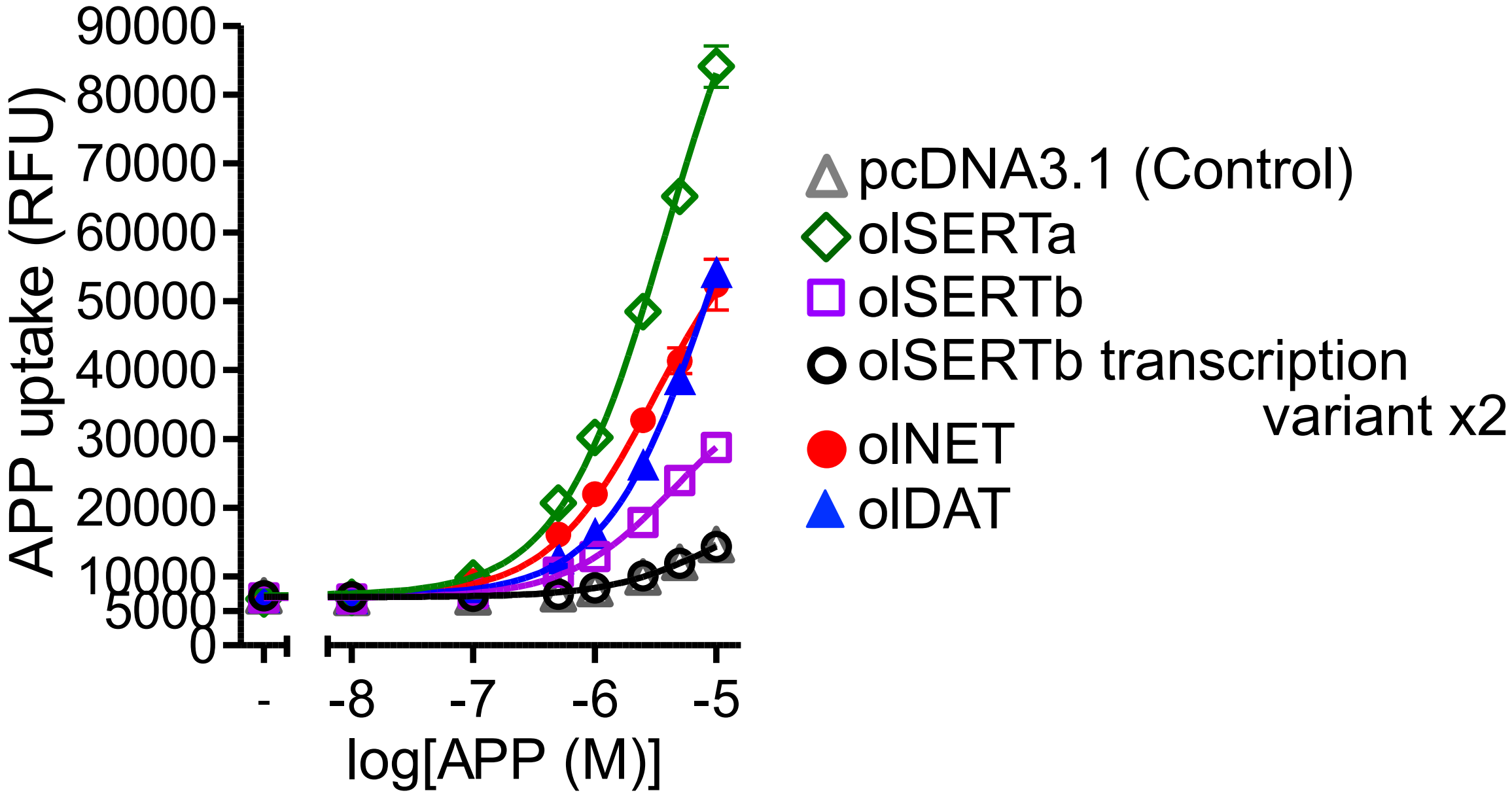

APP uptake (RFU)
90000
80000
70000
60000
50000
40000
30000
20000
10000
5000
0
-
-8
-7
-6
-5
log[APP (M)]
pcDNA3.1 (Control)
olSERTa
olSERTb
olSERTb transcription variant x2
olNET
olDAT

Fig. S6

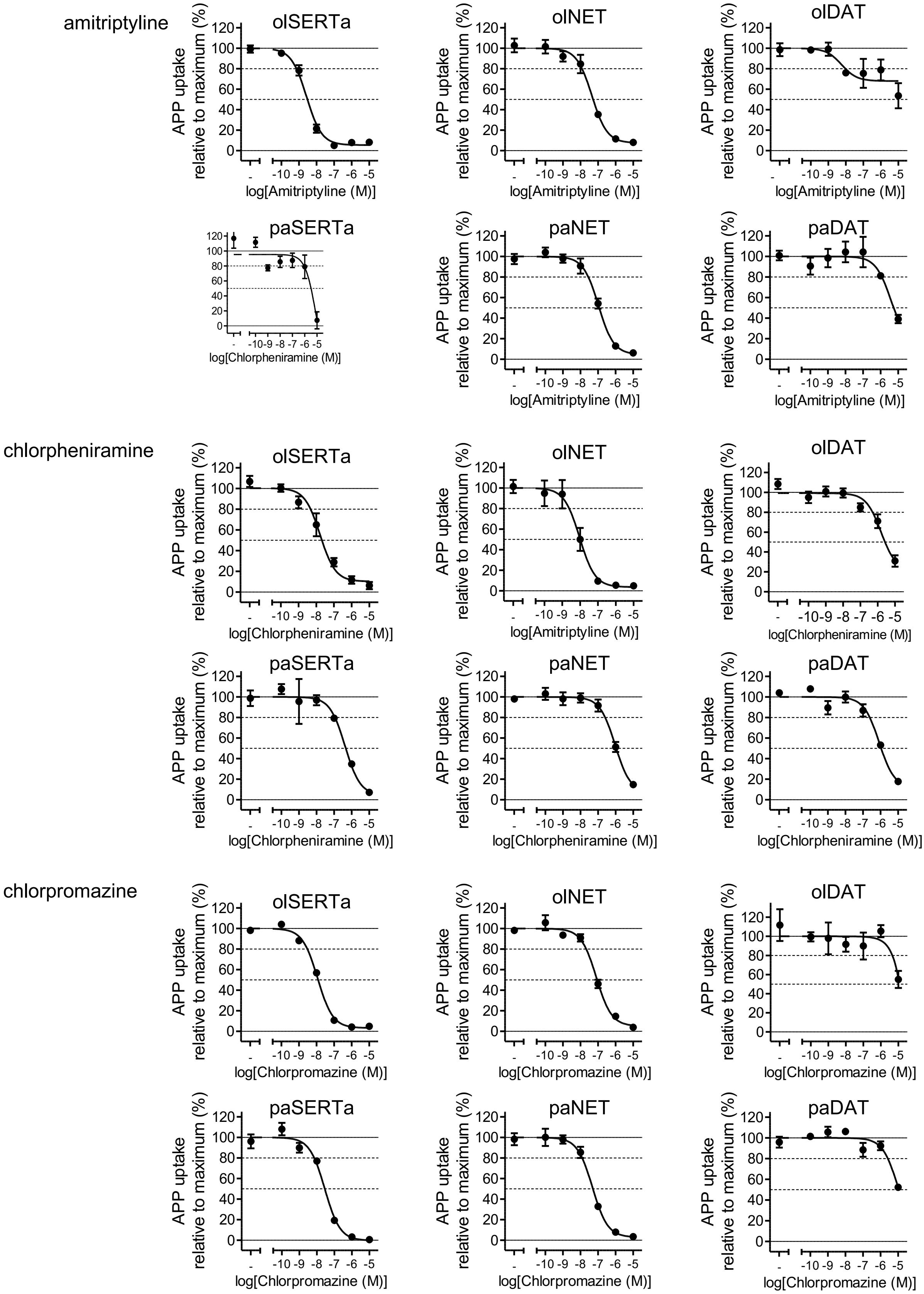

amitriptyline
olSERTa
olNET
olDAT
paSERTa
paNET
paDAT
APP uptake relative to maximum (%)
log[Amitriptyline (M)]
log[Chlorpheniramine (M)]
chlorpheniramine
olSERTa
olNET
olDAT
paSERTa
paNET
paDAT
log[Chlorpheniramine (M)]
log[Amitriptyline (M)]
chlorpromazine
olSERTa
olNET
olDAT
paSERTa
paNET
paDAT
log[Chlorpromazine (M)]

Fig. S6 (continue)

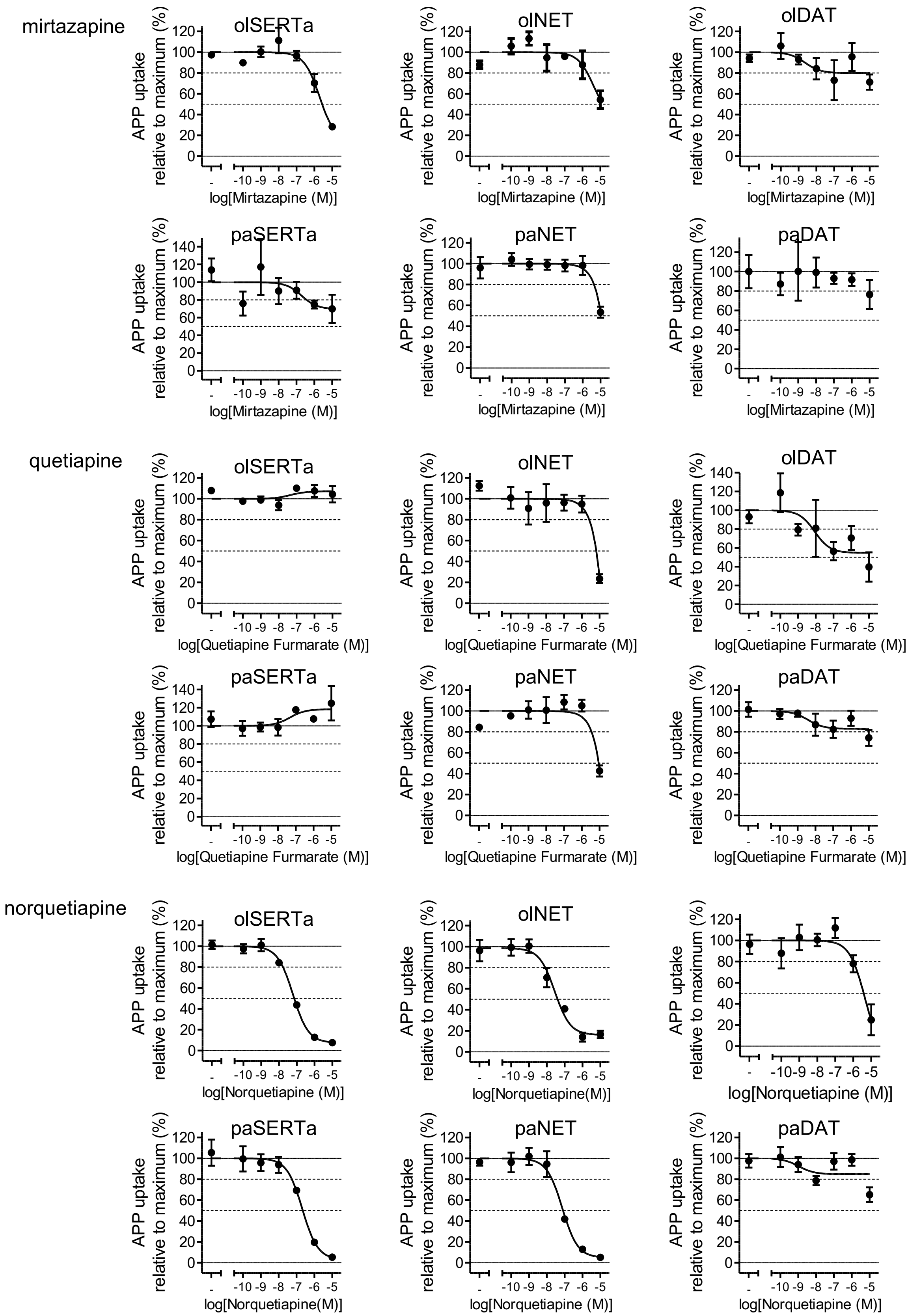

Fig. S6 (continue)

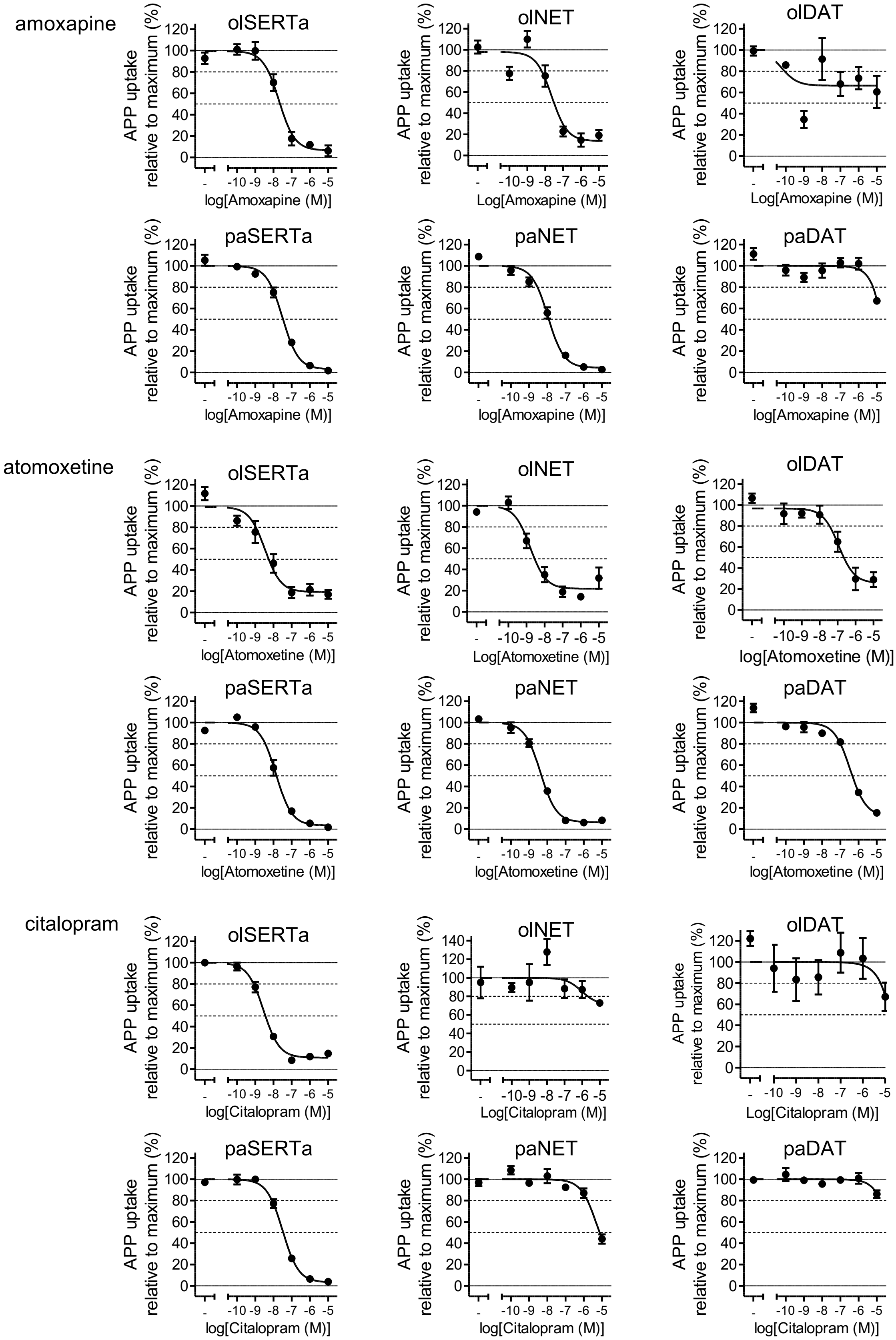

Fig. S6 (continue)

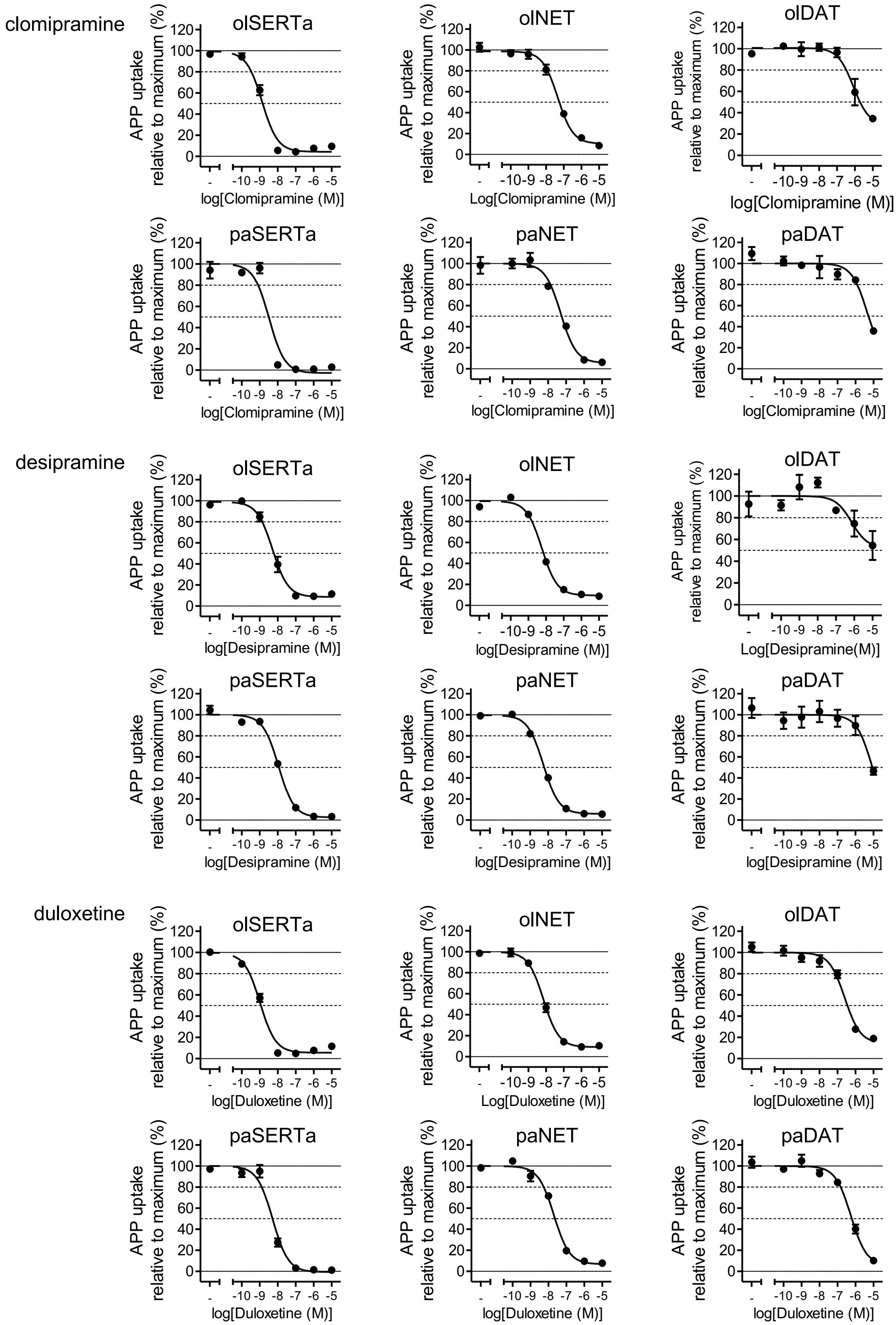

Fig. S6 (continue)

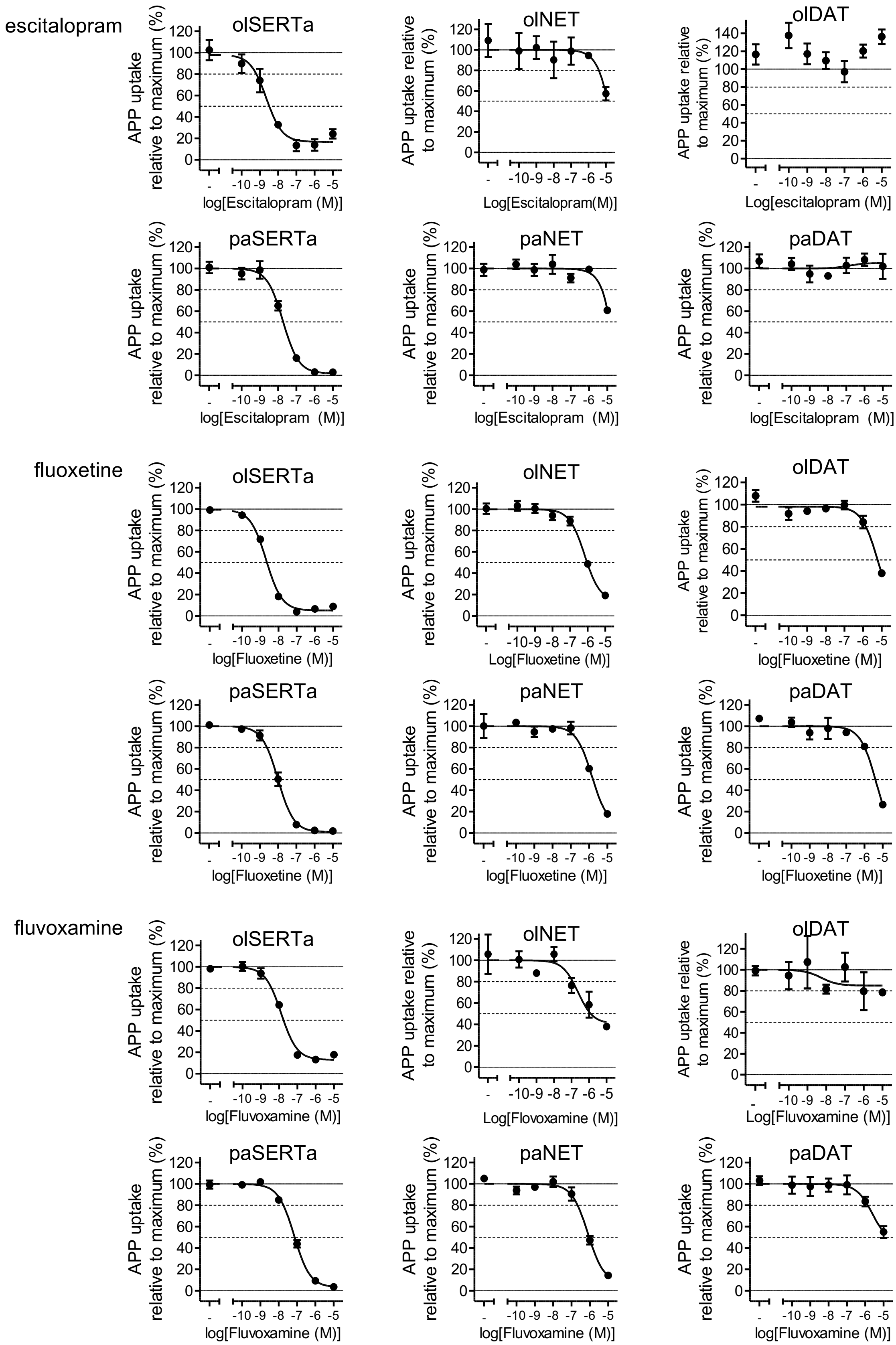

Fig. S6 (continue)

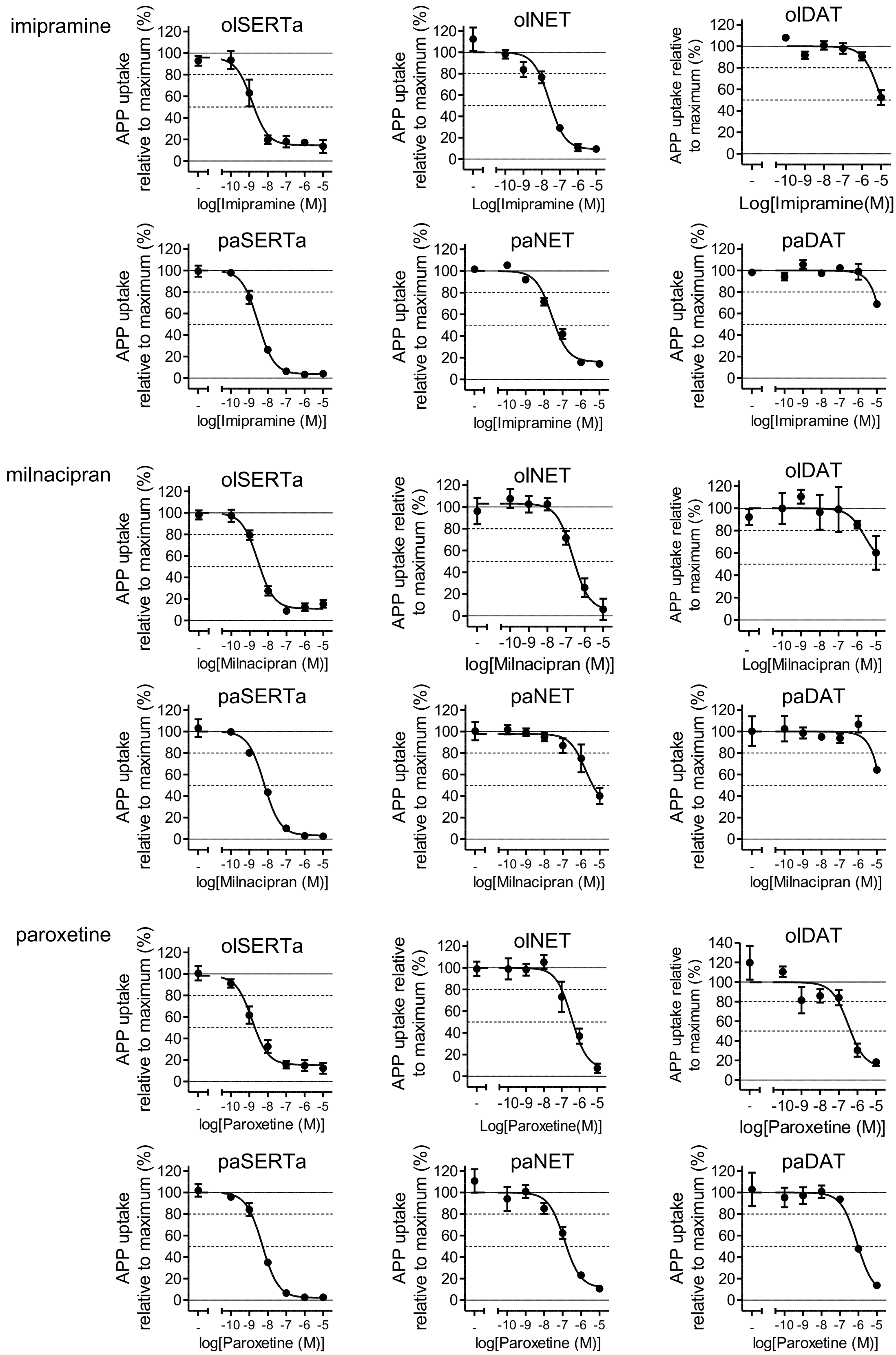

Fig. S6 (continue)

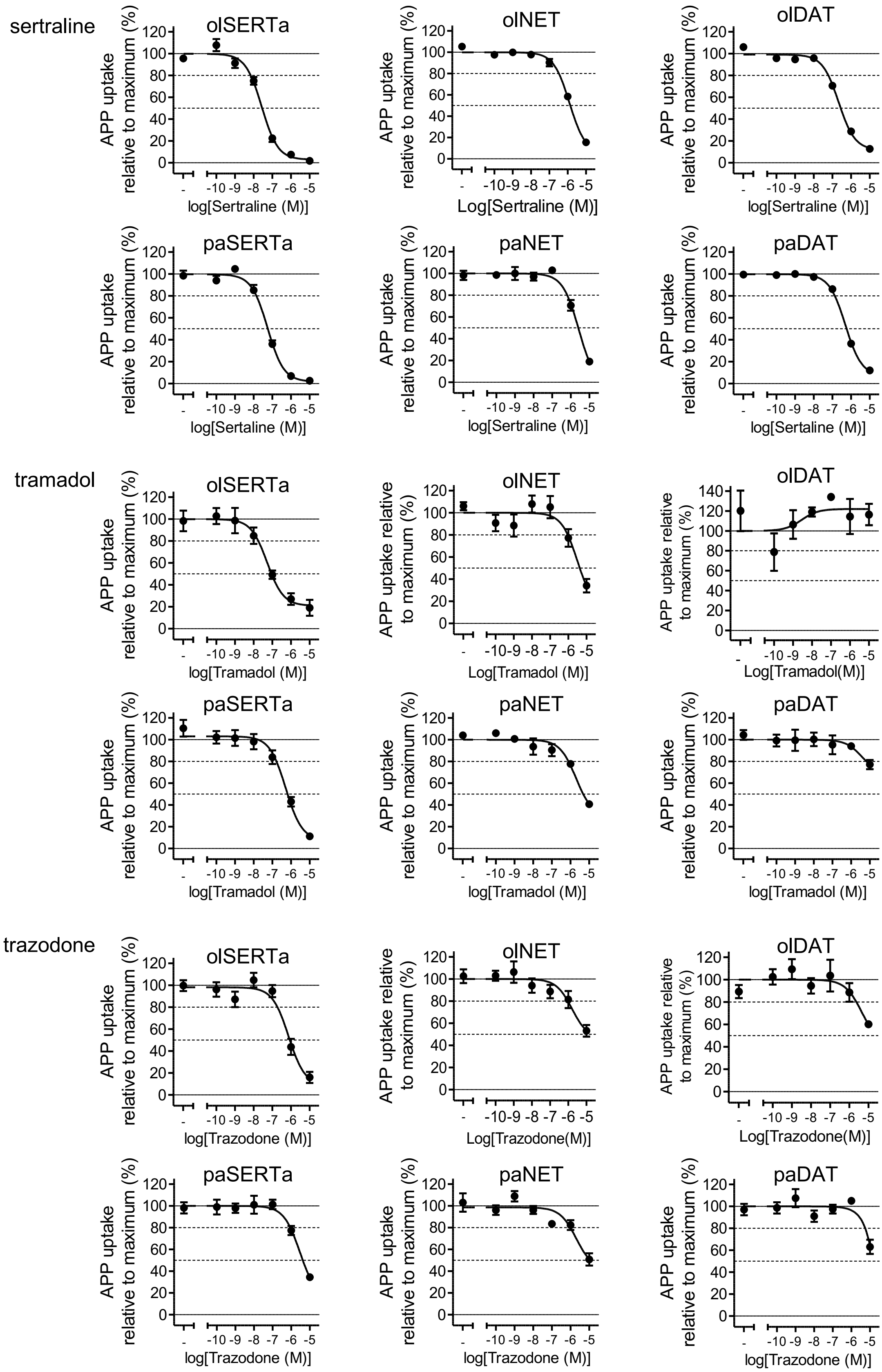

Fig. S6 (continue)

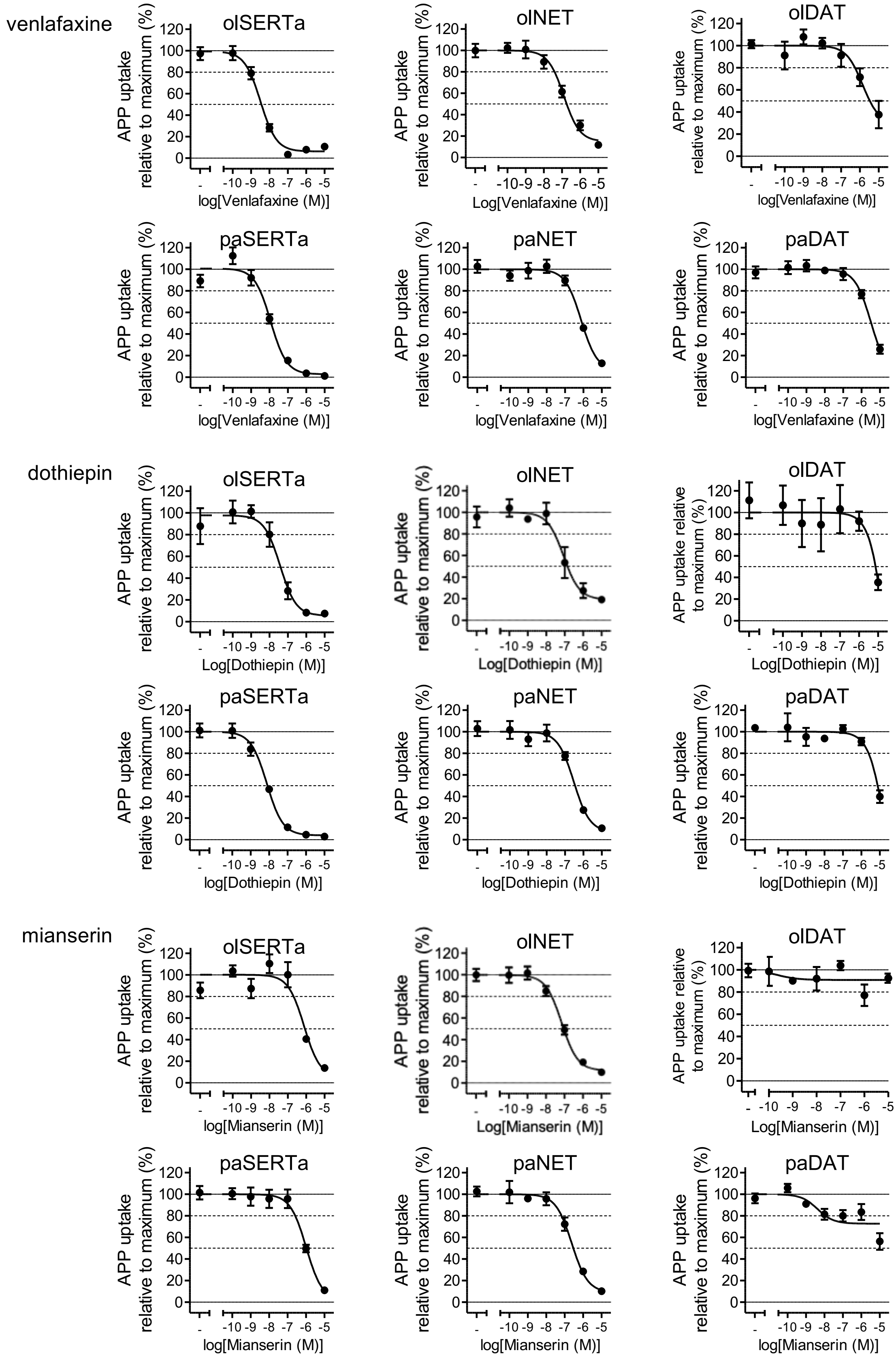

Fig. S6 (continue)

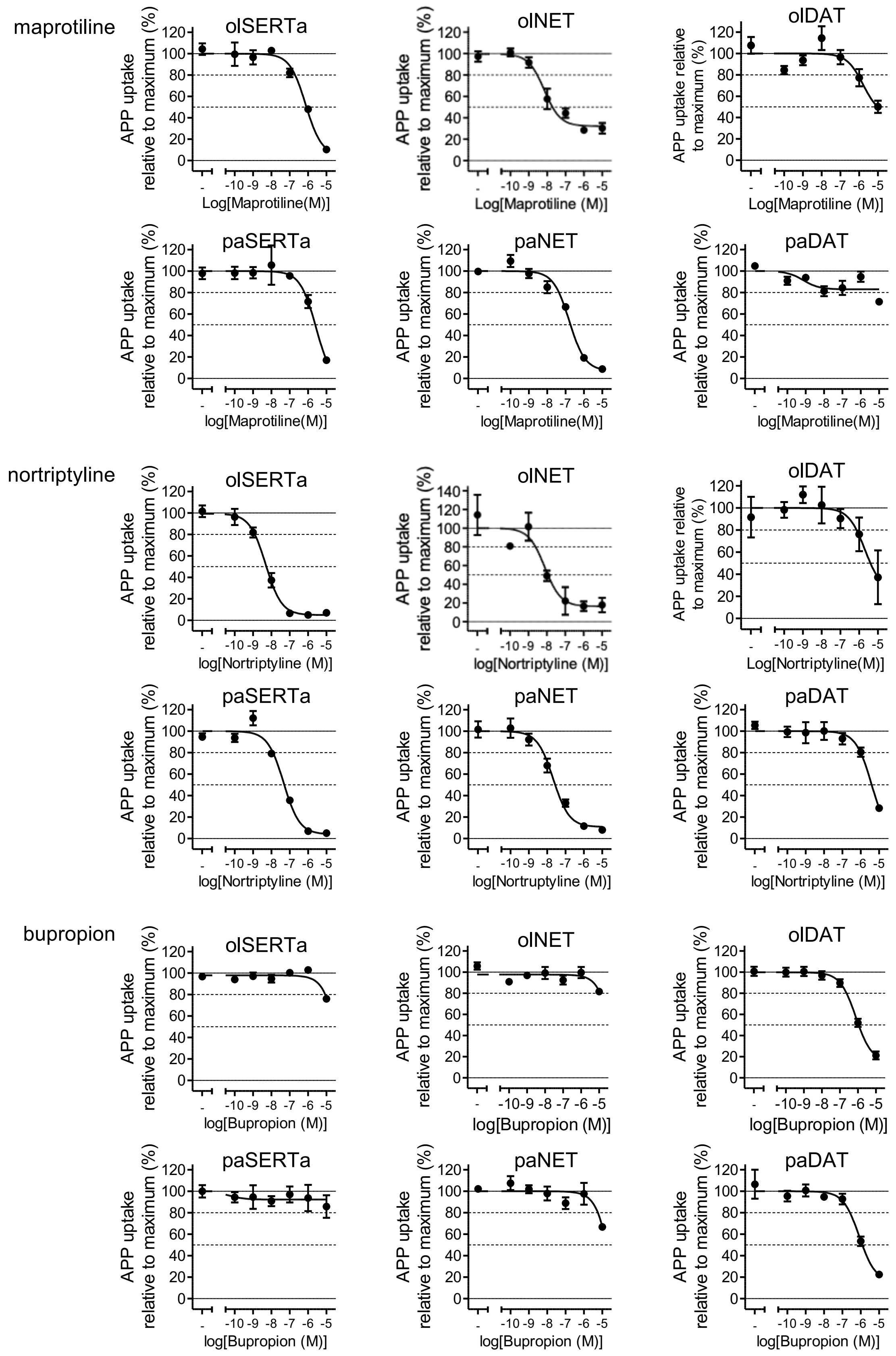

Fig. S7

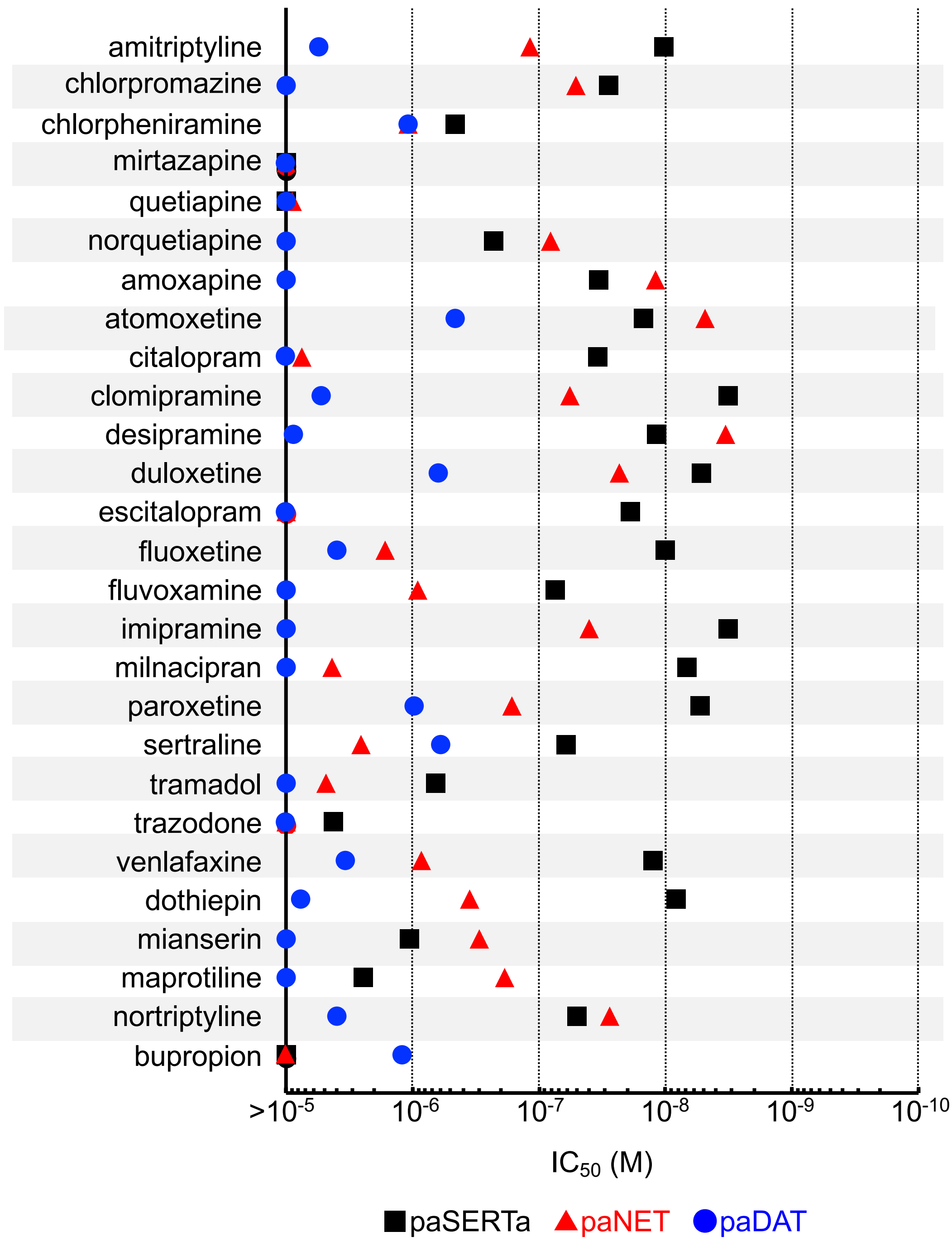

amitriptyline
chlorpromazine
chlorpheniramine
mirtazapine
quetiapine
norquetiapine
amoxapine
atomoxetine
citalopram
clomipramine
desipramine
duloxetine
escitalopram
fluoxetine
fluvoxamine
imipramine
milnacipran
paroxetine
sertraline
tramadol
trazodone
venlafaxine
dothiepin
mianserin
maprotiline
nortriptyline
bupropion
>10^-5
10^-6
10^-7
10^-8
10^-9
10^-10
IC50 (M)
paSERTa
paNET
paDAT

Fig. S8

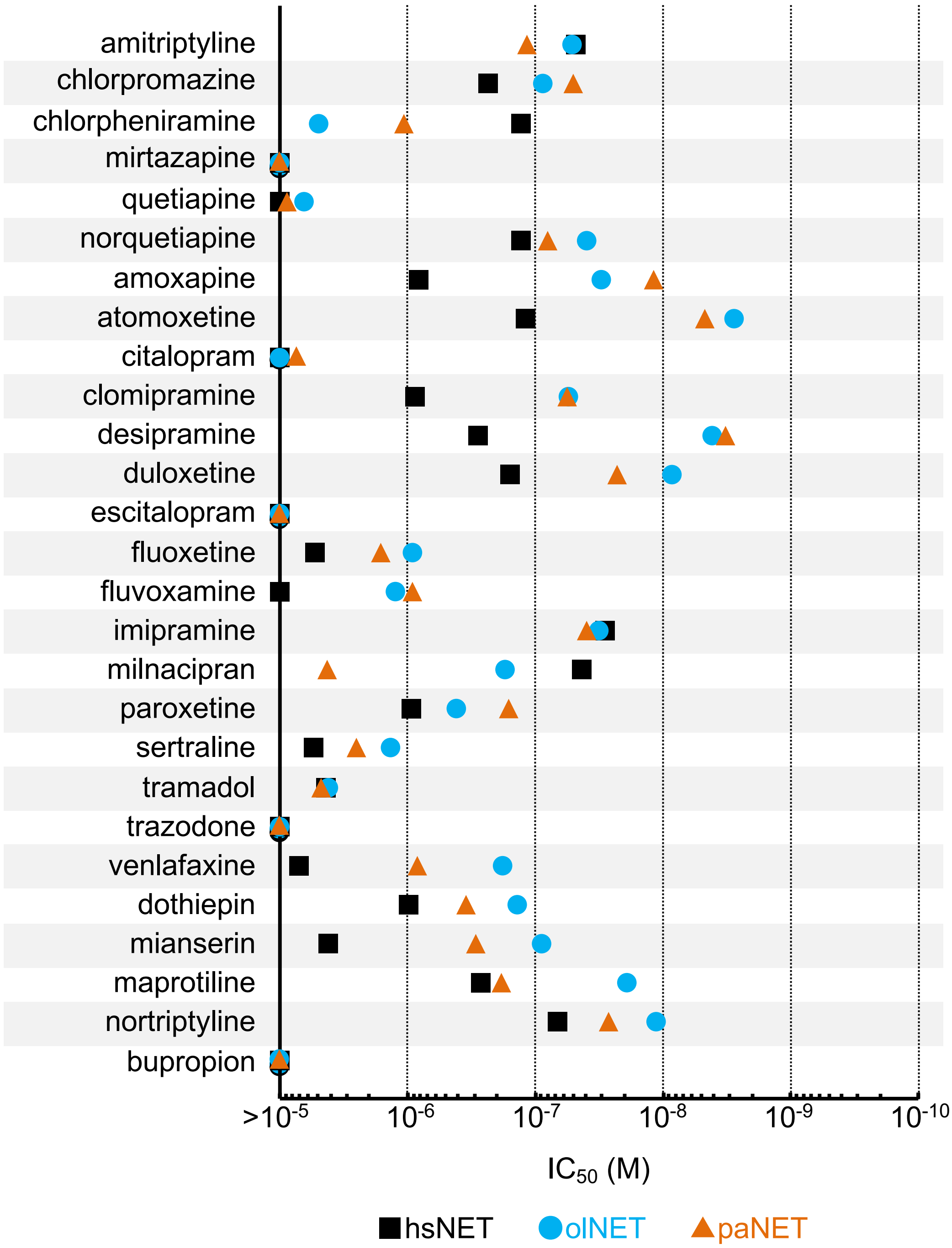

amitriptyline
chlorpromazine
chlorpheniramine
mirtazapine
quetiapine
norquetiapine
amoxapine
atomoxetine
citalopram
clomipramine
desipramine
duloxetine
escitalopram
fluoxetine
fluvoxamine
imipramine
milnacipran
paroxetine
sertraline
tramadol
trazodone
venlafaxine
dothiepin
mianserin
maprotiline
nortriptyline
bupropion
>10-5
10-6
10-7
10-8
10-9
10-10
IC50 (M)
hsNET
olNET
paNET

Fig. S9

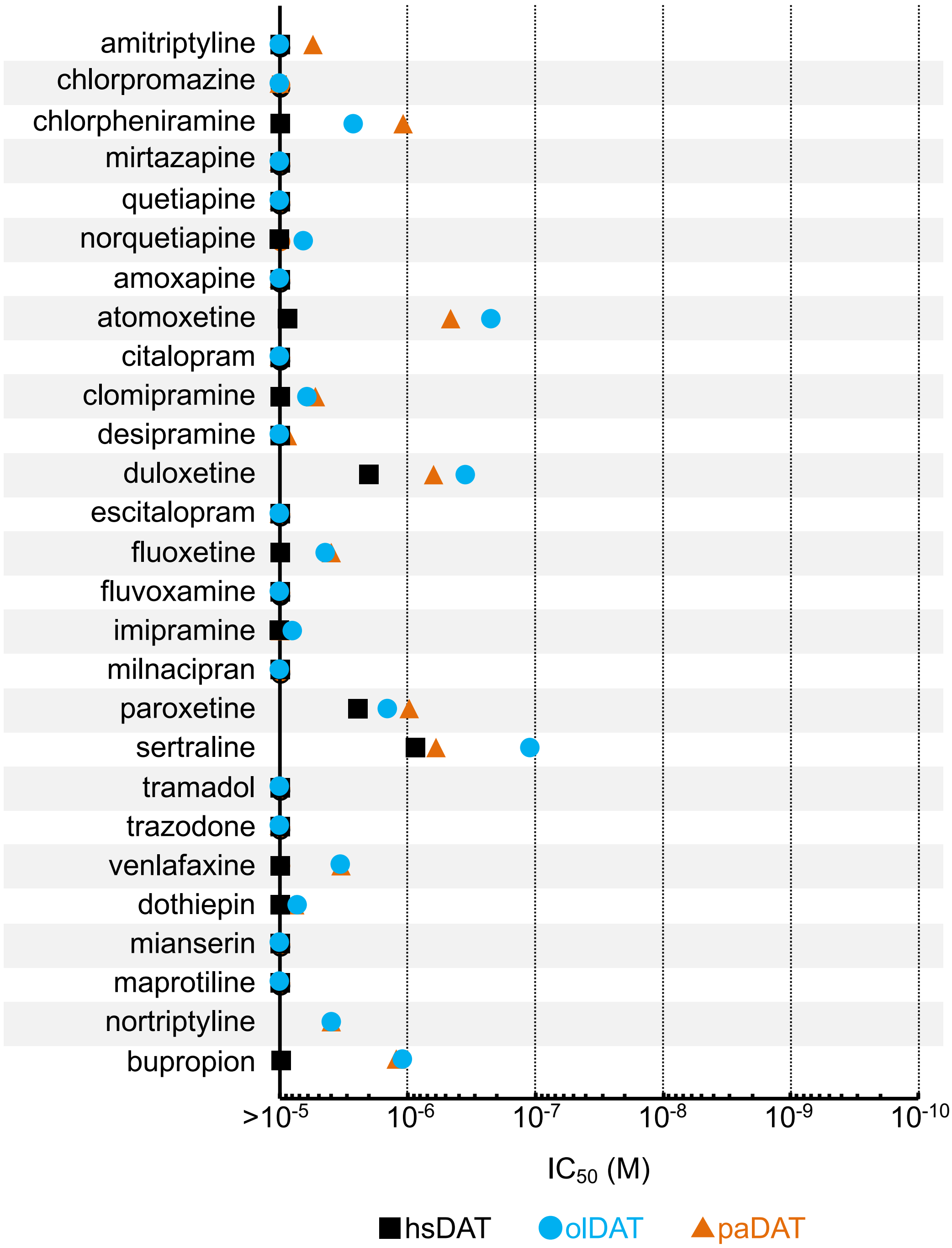
amitriptyline
chlorpromazine
chlorpheniramine
mirtazapine
quetiapine
norquetiapine
amoxapine
atomoxetine
citalopram
clomipramine
desipramine
duloxetine
escitalopram
fluoxetine
fluvoxamine
imipramine
milnacipran
paroxetine
sertraline
tramadol
trazodone
venlafaxine
dothiepin
mianserin
maprotiline
nortriptyline
bupropion
>10-5
10-6
10-7
10-8
10-9
10-10
IC50 (M)
hsDAT
olDAT
paDAT

Table S1

Supplemental Table S1. Sequence of primers for cloning

Gene	Animal		Primer		Purpose
SERTa	medaka	5'	ATCGACACACCTTGAGCCCTG	3'	gene cloning
		5'	GTGCTCTGCCCGTTTGGTCA	3'	
SERTbx1	medaka	5'	CTCTGACTGACTTTTCACAGTCCG	3'	gene cloning
		5'	ACACTGATGCGTTTGGCGCT	3'	
SERTbx2	medaka	5'	GTCTGCCCGGAAACATCACT	3'	gene cloning
		5'	ACACTGATGCGTTTGGCGCT	3'	
DAT	medaka	5'	CTTCAAAGGAAATCCCTGCTGAAGAG	3'	gene cloning
		5'	GCTCTGTTCTGTTCTTTGCAGCTT	3'	
NET	medaka	5'	TTTGTACGCATCCCAGACACG	3'	gene cloning
		5'	GGCGATCTGGGCTTCTATTTTCA	3'	
SERTa	ayu	5'	AAGCTTCAACCACCAGCCGCATTGCT	3'	gene cloning
		5'	CTCGAGAAGAGGGAGACAGCCCCCAG	3'	
SERTb	ayu	5'	GGTACCTGACTTTTGATAGTCCGCCAACC	3'	gene cloning
		5'	CTCGAGATGGTCCTCTTCAGGAACAGGAA	3'	
DAT	ayu	5'	GGATCCAGTGCCCCCCATGCTCAGAG	3'	gene cloning
		5'	CTCGAGCAGTCCTTCATCTTACGGCAGAAG	3'	
NET	ayu	5'	GGATCCGCTTCGCTCTACCGTGAAGATG	3'	gene cloning
		5'	CTCGAGGAATGTTGGGCAGCCATTG	3'	
			underbars indicate the sequence of restriction enzymes		
SERTa (for ISH)	medaka	5'	ATCGACACACCTTGAGCCCTG	3'	RNA probe preparation
		5'	GTGCTCTGCCCGTTTGGTCA	3'	
SERTb (for ISH)	medaka	5'	CCCAGATCAGCTCTCACACA	3'	RNA probe preparation
		5'	CAGCATGGATGTCTGGAATG	3'	
DAT (for ISH)	medaka	5'	CTTCAAAGGAAATCCCTGCTGAAGAG	3'	RNA probe preparation
		5'	GCTCTGTTCTGTTCTTTGCAGCTT	3'	
NET (for ISH)	medaka	5'	TTTGTACGCATCCCAGACACG	3'	RNA probe preparation
		5'	GGCGATCTGGGCTTCTATTTTCA	3'	

Table S2

Supplemental Table S2. Accession numbers of the sequences used in the phylogenetic analysis

Scientific Name	Common Name	Sequence Name	Genebank accession number	
Oryzias latipes)	medaka	SERTa	XP_011481223	glutamate to glycine at position 18
		SERTb	XP_004072650	
		SERTb transcription variant x2	XP_023814240	
		DAT	XP_020565684	
		NET	XP_004067359	alanine to proline at position 50, glutamate to glutamine at position 236, valine to leucine at position 250, and histidine to cysteine at position 604
Plecoglossus altivelis	ayu	SERTa	LC843755	
		SERTb	LC843756	
		DAT	LC843757	
		NET	LC843758	
Homo sapiens	human	SERT	NP_001036	
		DAT	NP_001035	
		NET	NP_001034	
Danio rerio	zebrafish	DAT	NP_571830	
		NET	XP_009301625	
		SERTa	NP_001035061	
		SERTax2	XP_009289948	
		SERTb	NP_001170930	
Branchiostoma floridae	Florida amphioxus	SERT-like	XP_035691751	
		NET-like	XP_035678786	
Ciona intestinalis	ascidian	SERT-like	XP_018669372	
Petromyzon marinus	sea lamprey	SERT-like1	XP_032823751	temporarily named SERT-like1
		SERT-like2	XP_032802967	temporarily named SERT-like2
		NET-like	XP_032826954	
		DAT		We did not use lamprey DAT because only partial sequence was registered on Genbank (XP_032834816) .
Latimeria chalumnae	coelacanth	SERT	XP_005999721	
		SERT-like	XP_005999512	
		DAT	XP_006011518	
		NET	XP_006000314	
Protopterus annectens	West African lungfish	SERT	XP_043910410	
		SERT-like	XP_043925691	
		DAT	XP_043921076	
		NET	XP_043937937	
Rhincodon typus	whale shark	SERTa	XP_048468710	
		SERTb	XP_020387137	
		DAT-like	XP_048464625	
		NET	XP_048461709	
Polypterus senegalus	gray bichir	SERTa	XP_039613104	
		SERTb	XP_039628723	
		DAT	XP_039593278	
		NET	XP_039618861	
Lepisosteus oculatus	spotted gar	SERT	XP_015222840	
		SERT-like	XP_006640395	
		DAT	XP_006635943	
		NET	XP_015224153	

Table S2 (continue)

Scleropages formosus	Asian arowana	SERT-like1	XP_018603757	temporarily named SERT-like1
		SERT-like2	XP_018604488	temporarily named SERT-like2
		SERT-like3	XP_018586211	temporarily named SERT-like3
		DAT	XP_018618351	
		NET1	XP_029108263	temporarily named NET1
		NET2	XP_029109498	temporarily named NET2, splice variant (XP_029109497)
Anguilla anguilla	European eel	SERTa	XP_035290712	
		SERTb	XP_035292066	
		DAT	XP_035259108	
		NET-like1	XP_035250904	temporarily named NET-like1
		NET-like2	XP_035274205	temporarily named NET-like2
Gasterosteus aculeatus	Stickleback	SERTa	XP_040045414	
		SERTb	XP_040052758	
		DAT	XP_040021224	
		NET	XP_040020457	
Kryptolebias marmoratus	mangrove killifish	SERTa	XP_003975030	
		SERTb	XP_017269392	
		DAT	XP_017279121	
		NET	XP_017265289	
Takifugu rubripes	Japanese pufferfish	SERT	XP_029699195	
		SERT-like	XP_003975030	
		DAT	XP_029694770	
		NET	P_011607998	
Xenopus tropicalis	tropical clawed frog	SERT1	XP_017946918	temporarily named SERT1
		SERT2	XP_012825214	temporarily named SERT2
		DAT	XP_002933174	
		NET	XP_017948962	
Rhinatrema bivittatum	two-lined caecilian	SERT	XP_029467067	
		SERT-like	XP_029427205	
		NET	XP_029464437	
Eublepharis macularius	leopard gecko	SERT	XP_054857638	
		SERT-like	XP_054851861	
		NET	XP_054856245	
Alligator mississippiensis	alligator	SERT	XP_006271355	
		SERT-like	XP_006272897	temporarily named SIRT-like
		NET	XP_014458861	
Gallus gallus	chicken	SERT	NP_998737	
		SERT-like	XP_040540149	
		NET	NP_990047	

Table S3

Supplemental Table S3. List of test chemicals used in this study

chemical	CAS number	purity	manufacturer
amitriptyline hydrochloride	549-18-8	>98%	Sigma-Aldrich (St. Louis, MO, USA)
chlorpromazine hydrochloride	69-09-0	>98%	Sigma-Aldrich (St. Louis, MO, USA)
chlorpheniramine maleate	113-92-8	>98%	LKT Laboratories (St. Paul, Minnesota, USA)
mirtazapine	85650-52-8	>98%	Tokyo Chemical Industry (Tokyo, Japan)
quetiapine fumarate	111974-72-2	>98%	Fujifilm Wako Pure Chemical (Osaka, Japan)
norquetiapine hydrochloride	753475-15-9	>98%	Sigma-Aldrich (St. Louis, MO, USA)
amoxapine	14028-44-5	>98%	Fujifilm Wako Pure Chemical (Osaka, Japan)
atomoxetine hydrochloride	82248-59-7	>98%	Tokyo Chemical Industry (Tokyo, Japan)
citalopram hydrobromide	59729-32-7	>98%	LKT Laboratories (St. Paul, Minnesota, USA)
clomipramine hydrochloride	17321-77-6	>98%	Fujifilm Wako Pure Chemical (Osaka, Japan)
desipramine hydrochloride	58-28-6	>98%	Fujifilm Wako Pure Chemical (Osaka, Japan)
duloxetine hydrochloride	136434-34-9	>98%	Fujifilm Wako Pure Chemical (Osaka, Japan)
escitalopram oxalate	219861-08-2	>98%	Fujifilm Wako Pure Chemical (Osaka, Japan)
fluoxetine hydrochloride	56296-78-7	>98%	LKT Laboratories (St. Paul, Minnesota, USA)
fluvoxamine	54739-18-3	>98%	Toronto Research Chemicals (North York, Canada)
imipramine hydrochloride	113-52-0	>98%	Fujifilm Wako Pure Chemical (Osaka, Japan)
milnacipran hydrochloride	101152-94-7	>98%	Fujifilm Wako Pure Chemical (Osaka, Japan)
paroxetine hydrochloride	78246-49-8	>98%	Fujifilm Wako Pure Chemical (Osaka, Japan)
sertraline hydrochloride	79559-97-0	>98%	Fujifilm Wako Pure Chemical (Osaka, Japan)
tramadol	36282-47-0	>98%	Sigma-Aldrich (St. Louis, MO, USA)
trazodone	25332-39-2	>98%	Sigma-Aldrich (St. Louis, MO, USA)
venlafaxine hydrochloride	99300-78-4	>98%	Fujifilm Wako Pure Chemical (Osaka, Japan)
dothiepin	113-53-1	>98%	Toronto Research Chemicals (North York, Canada)
mianserin hydrochloride	21535-47-7	>98%	Tokyo Chemical Industry (Tokyo, Japan)
maprotiline hydrochloride	10347-81-6	>98%	Tokyo Chemical Industry (Tokyo, Japan)
nortriptyline hydrochloride	894-71-3	>98%	Tokyo Chemical Industry (Tokyo, Japan)
bupropion hydrochloride	31677-93-7	>98%	Fujifilm Wako Pure Chemical (Osaka, Japan)
